# Reverse engineering of logic-based differential equation models using a mixed-integer dynamic optimization approach

**DOI:** 10.1093/bioinformatics/btv314

**Published:** 2015-05-21

**Authors:** David Henriques, Miguel Rocha, Julio Saez-Rodriguez, Julio R. Banga

**Affiliations:** ^1^Bioprocess Engineering Group, Spanish National Research Council, IIM-CSIC, C/Eduardo Cabello 6, 36208 Vigo, Spain, ^2^Centre of Biological Engineering, University of Minho, Campus de Gualtar, 4710-057 Braga, Portugal and ^3^European Molecular Biology Laboratory, European Bioinformatics Institute, Wellcome Trust Genome Campus, Cambridge, UK

## Abstract

**Motivation:** Systems biology models can be used to test new hypotheses formulated on the basis of previous knowledge or new experimental data, contradictory with a previously existing model. New hypotheses often come in the shape of a set of possible regulatory mechanisms. This search is usually not limited to finding a single regulation link, but rather a combination of links subject to great uncertainty or no information about the kinetic parameters.

**Results:** In this work, we combine a logic-based formalism, to describe all the possible regulatory structures for a given dynamic model of a pathway, with mixed-integer dynamic optimization (MIDO). This framework aims to simultaneously identify the regulatory structure (represented by binary parameters) and the real-valued parameters that are consistent with the available experimental data, resulting in a logic-based differential equation model. The alternative to this would be to perform real-valued parameter estimation for each possible model structure, which is not tractable for models of the size presented in this work. The performance of the method presented here is illustrated with several case studies: a synthetic pathway problem of signaling regulation, a two-component signal transduction pathway in bacterial homeostasis, and a signaling network in liver cancer cells.

**Supplementary information:** Supplementary data are available at *Bioinformatics* online.

**Contact:** julio@iim.csic.es or saezrodriguez@ebi.ac.uk

## 1 Introduction

In recent years, there has been a growing interest in the application of logic formalisms to systems biology, and in particular to model signal transduction (Albert and Thakar, 2014; Samaga and Klamt, 2013). The basis of this model formalism lies in the assumption that cells process information of certain stimuli approximately by logic circuits, and their simplicity makes them particularly amenable to model large networks and integrate pathway knowledge from databases with high-throughput data (Blinov and Moraru, 2012).

Logic models were first introduced by Kauffman (1969) to model gene regulatory networks. Since then, diverse modifications from the original formalism were developed. In particular various extensions have been developed to accommodate continuous values (e.g. Aldridge *et al.*, 2009; Bernardo-Faura *et al.*, 2014; Bonneau *et al.*, 2006, de Jong, 2002; Mendoza and Xenarios, 2006). Amongst these formalisms, logic-based ordinary differential equations (ODEs) are well suited to handle time series in a precise manner. The main idea is to transform the logic model into a continuous homologue in the form of ODEs. Since it is based on a logic circuit, this formalism does not require information about the biochemistry (e.g. stoichiometry or type of kinetics), and at the same time, since it provides a model of differential equations, we can accurately perform dynamic simulations for the state variables trajectories. Several methods have been proposed in the literature to transform Boolean logic model into ODE approximations (Bonneau *et al.*, 2006; Mendoza and Xenarios, 2006). CellNOpt, relies in multivariate polynomial interpolation introduced by Wittmann *et al.* (2009).

Logic formalisms have been used to reverse engineer biochemical networks from data, i.e*.* to obtain a mechanistic dynamic model from time-series data. One early example is the work by Akutsu *et al.* (1999) which proposed a brute force approach that infers the Boolean function of a few top *k* regulators, node by node. Other methods treat these networks in a global manner (instead of fitting logic functions node by node) borrowing ideas from optimization and machine learning to avoid excessive model complexity (Bonneau *et al.*, 2006; Saez-Rodriguez *et al.*, 2009). In Saez-Rodriguez *et al.* (2009) networks derived from of prior knowledge, from e.g. public repositories of manually curated networks, are expanded into a hypergraph, where all the possible logic gates are represented and optimization strategies are used to find which networks could best reproduce the data with the smallest number of hyperedges. This method is implemented in the software CellNOpt (Terfve *et al.*, 2012) for various logic formalisms and is designed to reverse engineer Boolean models, mainly in a protein signaling environment, given data from perturbation experiments.

Here, we present a mixed-integer global optimization approach for the problem of reverse engineering signaling and regulatory networks as logic-based ODEs from a source of prior-knowledge containing multiple possible regulation links and experimental data. The problem of identifying the logic gates is formulated as a simultaneous model selection and parameter identification problem. From the optimization point of view, this corresponds to a mixed integer dynamic optimization (MIDO) problem. Although MIDO problems are typically hard, we show that solutions can be achieved for rather complex networks by applying global optimization meta-heuristics.

Only a few authors have considered the use of mixed-integer nonlinear programming for reverse engineering purposes. Sambo *et al.* (2012) proposed the algorithm mixed optimization for reverse engineering (MORE), which consists in a bi-level optimization where the discrete (binary) level communicates with the continuous (NLP) level and *vice versa*. For model representation, a structured formalism, formally identical to dynamic recurrent neural networks, is used. Guillén-Gosálbez *et al.* (2013) have presented a deterministic method for identification of regulatory structure and kinetic parameters in biochemical networks, transforming the MIDO problem into an approximated large-scale MINLP, which was then solved by a nonlinear branch and bound method. To avoid local minima the authors provided high quality initial solutions to the solver. These solutions were obtained by solving a set of relaxed problems from different starting points. Despite these advances, the major drawback of deterministic global methods is that the computational effort increases very rapidly with problem size. More recently, Rodriguez-Fernandez *et al.* (2013) have shown how to apply mixed integer nonlinear programming (MINLP) to perform simultaneous model discrimination and parameter estimation in dynamic models of cellular systems.

This paper is organized as follows: first, we present the formulation of the mixed-integer dynamic optimization problem making use of logic-based dynamic models. Then we present a solution strategy based on global optimization metaheuristics. Next, the performance and capabilities of the new approach are illustrated with several reverse engineering case studies: a synthetic pathway of signaling regulation, a signal transduction pathway in bacterial homeostasis, and a signaling pathway in live cancer cells. Finally, the main conclusions are outlined.

## 2 Methods

### 2.1 Logic-based ordinary differential equation models

Logic models describe the flow of information inside the cell by means of discrete states (logic decisions) that can assume either the values 0 or 1. Each state $x_{i}\in \{0,1\}$ is, therefore, represented by a binary variable that is systematically updated according to a Boolean function $B_{i}(x_{i1},x_{i2},...,x_{iN})\in \{0,1\}$ of its *N* inputs (*x_ij_*). As an example, consider the case where a specific protein is to be phosphorylated in two sites by different kinases, and both phosphorylations are required to activate the protein. This can be modeled as a logic conjunction (AND gate). In contrast, if two different kinases can phosphorylate the same site activating the propagation of the downstream signaling independently, this can be regarded as a logic disjunction (OR gate). Furthermore, if a signal inhibits the propagation of another one, this can be depicted as a negation (NOT gate).

If one uses only AND/OR/NOT gates, logic models can be represented using a hypergraph structure (incidence matrix). In this case, a hyperedge with more than one input represents and AND gate, and OR gates are encoded by multiple hyperedges arriving at a given node. The idea in logic-based ODE models is to convert each Boolean update function into a continuous homologue ${\bar{B}}_{i}\in [0,1]$, where the species ${\bar{x}}_{i}\in [0,1]$ is allowed to take continuous values between 0 and 1, and its temporal behavior is described by:

(1) $${\dot{\bar{x}}}_{i}=\frac{1}{\tau_{i}}\cdot ({\bar{B}}_{i}({\bar{x}}_{i1},{\bar{x}}_{i2},...,{\bar{x}}_{ij})-{\bar{x}}_{i})$$

where $\tau_{i}$ can be interpreted as the life-time of the species $x_{i}$.

In order to achieve a continuous homologue, Wittmann *et al.* (2009) introduced HillCubes. These functions are based on multivariate polynomial interpolation and incorporate Hill kinetics, which are known to provide a good generalized approximation of the synergistic dynamics of gene regulation.

To obtain HillCubes, a first transformation method is required to reach a continuous homologue from the Boolean update function. Table 1 provides an example on how an OR gate would be transformed into a BoolCube (${\bar{B}}^{I}$), obtained by multi-linearly interpolating the Boolean update function:

(2) $$\begin{array}{l} {\bar{B}}^{I}({\bar{x}}_{1},\ldots ,{\bar{x}}_{N})\\ \;=\sum_{x_{1}=0}^{1}\ldots \sum_{x_{N}=0}^{1}[B(x_{1},...,x_{N})\cdot \prod_{i=1}^{N}(x_{i}{\bar{x}}_{i}+[1-x_{i}][1-{\bar{x}}_{i}])] \end{array}$$

**Table 1.** The relationship between the OR Boolean update function $B(x_{1},x_{2})$ and its continuous homologue ${\bar{B}}^{I}({\bar{x}}_{1},{\bar{x}}_{2})$, obtained by multivariate polynomial interpolation (Wittmann *et al.*, 2009), is illustrated with the help of a truth table

$x_{1}$	$x_{2}$	$B(x_{1},x_{2})$	${\bar{B}}^{I}({\bar{x}}_{1},{\bar{x}}_{2})=...$
0	0	0	$0\cdot (1-{\bar{x}}_{1})\cdot (1-{\bar{x}}_{2})+$
0	1	1	$1\cdot (1-{\bar{x}}_{1})\cdot {\bar{x}}_{2}+$
1	0	1	$1\cdot {\bar{x}}_{1}\cdot (1-{\bar{x}}_{2})+$
1	1	1	$1\cdot {\bar{x}}_{1}\cdot {\bar{x}}_{2}$

Note: For every combination of the Boolean variables *x*_1_ and *x*_2_, a term is added to ${\bar{B}}^{I}({\bar{x}}_{1},{\bar{x}}_{1}$) depending on $B(x_{1},x_{2})$.

BooleCubes are accurate homologues of Boolean functions; however, these fail to represent the typical sigmoid shape switch-like behavior, often present in molecular interactions (Krumsiek *et al.*, 2010). The latter can be achieved by replacing the ${\bar{x}}_{i}$ by a Hill function:

(3) $$f^{H}({\bar{x}}_{i})=\frac{{\bar{x}}_{i}^{n}}{{\bar{x}}_{i}^{n}+k^{n}}$$

or the normalized Hill function:

(4) $$f^{Hn}({\bar{x}}_{i})=\frac{f^{H}({\bar{x}}_{i})}{f^{H}(1)}$$

A further discussion about continuous homologues and methodology to obtain logic-based ODE models can be found in (Wittmann *et al.*, 2009).

### 2.2 Problem formulation

In order to find the logic gates which best describe the behavior of a given network, we will be interested in a formulation similar to what was used by Saez-Rodriguez *et al.* (2009) within a Boolean logic framework or Morris *et al.* (2011) within the constrained fuzzy-logic formalism. The idea here is that starting from a directed graph containing only the interactions and their signs (activating or inhibitory) we can obtain an expanded hypergraph containing all the possible gates where edges with two or more inputs (a hyperedge) represent a logical conjunction (AND gate) and single edges represent a logical disjunction (OR gate).

**Fig. 1.**
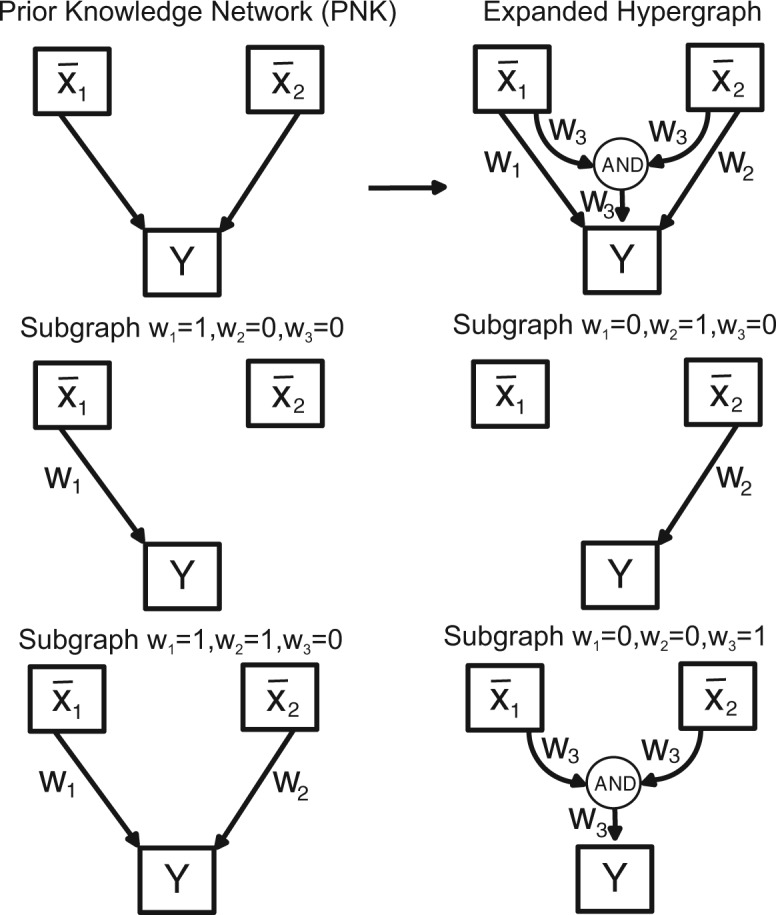
A simple PKN, the corresponding expanded hypergraph and a number of possible solutions for the obtained subgraph are shown to illustrate the association of the used weights (*w*) with each hyperedge. There are four options in this example: if *w*_1_ is equal to one, ${\bar{x}}_{1}$ activates *y*. If *w*_2_ is equal to one, ${\bar{x}}_{2}$ activates *y*. If both *w*_1_ and *w*_2_ are equal to one, *y* can be activated by ${\bar{x}}_{1}$ or ${\bar{x}}_{2}$. If *w*_3_ is equal to one and both *w*_1_ and *w*_2_ are zero, ${\bar{x}}_{1}$ and ${\bar{x}}_{2}$ are required to activate *y*. OR gates are implicitly represented as simple edges

The problem can be formulated as the following:

(5) minimizen,k,τ,w F=∑ϵ=1nϵ∑o=1noϵ∑s=1nsϵ,o(y∼sϵ,o−ysϵ,o)2subject to Esub={ei|wi=1}, i=1,…,nhyperedgesHsub=(V,Esub)LBn≤n≤UBnLBk≤k≤UBkLBτ≤τ≤UBτx¯˙=f(Hsub,x¯,n,k,τ,t)x¯(t0)=x¯0y=g(Hsub,x¯,n,k,τ,t)

where $H_{sub}$ is the subgraph containing only the hyperedges ($E_{sub}$), defined by the binary variables *w* (see Fig. 1). Additionally *n*, *k* and *τ* are the continuous parameters needed for the logic-based ODE approach. These parameters are limited by upper and lower bounds (e.g. LB*_k_*). The model dynamics ($\dot{\bar{x}}$) are given by the function *f*. This set of differential equations varies according to the subgraph (and therefore also according to the integer variables vector *w*). Finally, the system of differential equations has to be solved to obtain the simulated data. The objective function is the squared difference between the simulated data (*y*) and the experimental data ($\tilde{y}$) and our goal is to minimize this value for every experiment (*ϵ*), observed species (*o*) and sampling point (*s*). The simulation data *y* is given by an observation function *g* of the model dynamics at time *t*.

### 2.3 Solving the mixed integer dynamic optimization problem

The problem considered in this work belongs to the category of network reverse engineering, where the objective is to simultaneously determine network topology and continuous model parameters which explain a given set of data. The network contains a series of possible regulatory mechanisms and our goal is to find the set that best describes the data. Our dynamic formulation, shown in the previous section, makes use of logic-based ODEs. Essentially, the binary variables define the structure of the system of ODEs describing the dynamic behaviour. Additionally, a set of continuous parameters modulating those dynamics need to be estimated. From the optimization point of view, this problem belongs to the class of mixed integer dynamic optimization (MIDO).

In general, model calibration of a nonlinear dynamic model is a difficult task. Due to the nonlinear and constrained nature of the system dynamics, these problems are multi-modal (non-convex) (Banga, 2008; Villaverde and Banga, 2014). The MIDO considered here augments the difficulties of solving non-linear, non-convex problems with those typical of combinatorial problems.

MIDO problems can be solved using deterministic or stochastic global optimization methods. A broad overview of global optimization with a special focus on deterministic methods, including mixed-integer nonlinear programminng and the global optimization of dynamic systems, can be found in the works of Biegler and Grossmann (2004), Grossmann and Biegler (2004), Chachuat *et al.* (2005) and Houska and Chachuat (2014).

Regarding the specific usage of deterministic MIDO methods for systems identification, significant advances have been made recently, as shown by Guillén-Gosálbez *et al.* (2013). However, these still suffer from the major drawback of deterministic global methods, i.e*.* computational effort increases extremely rapidly with problem size.

Stochastic algorithms for global optimization cannot offer guarantees of global optimality, but usually converge to the vicinity of the global optimum in reasonable computation times, at least for small and medium scale problems. However, for larger problems their computational cost is very significant (Moles *et al.*, 2003). Hybrid approaches try to combine the best of the two worlds by combining global stochastic methods with efficient (local) deterministic optimization methods (Banga *et al.*, 2004; Rodriguez-Fernandez *et al.*, 2006a). In this context, metaheuristics (i.e*.* guided heuristics) have been particularly successful, ensuring the proper solution of these problems by adopting a global optimization approach, while keeping the computational effort under reasonable values thanks to efficient local optimization solvers (Rodriguez-Fernandez *et al.*, 2006b).

In this work, we have chosen a recent metaheuristic based on the combination of an enhanced scatter search (eSS) method as global solver (Egea *et al.*, 2010) with a Mixed-Integer Sequential Quadratic Programming (MISQP) (Exler *et al.*, 2012) local solver. eSS is an evolutionary algorithm for complex-process optimization that employs some elements of scatter search and path relinking. MISQP is a trust region sequential quadratic programming solver adapted to solve MINLP problems. In this code, instead of solving continuous quadratic programs, the solution is approximated by a series of mixed-integer convex quadratic programming problems. In addition, MISQP accepts black-box problems and, thus, does not require the problem to be transformed into an algebraic form, a typical requirement of most MINLP methods. As shown below, we compared the performance of eSS with two other modern metaheuristics, ACOmi (Ant-Colony for Mixed Integer) (Schlüter *et al.*, 2009) and MITS (Mixed-Integer Tabu Search) (Exler *et al.*, 2008). For the class of problems considered here, we found that eSS consistently provided the best results.

### 2.4 A multi-phase scatter search with relaxed MINLPs

The MIDO problem formulated above is extremely challenging to solve. Although the initial results obtained with the enhanced scatter search (eSS) method (Egea *et al.*, 2010) were promising, a second objective of this work was to improve the algorithm in terms of convergence speed while keeping robustness in order to ensure a good scale-up for realistic applications. For this purpose, we have devised a multi-phase scatter search (MPeSS) strategy which, in a first phase, computes intermediate solutions of relaxed MINLPs and, in a second phase, uses them as initial points for solving the original MINLP.

In order to reformulate a relaxed problem, we consider each hyperedge to be associated with a continuous weight instead of a binary variable. Each weight will appear as an additional term in its corresponding minterm from the truth table. When several weights affect a single minterm, then we can apply the multivariate polynomial interpolation of an OR gate. Table 2 and Figure 1 illustrate the problem formulation where variables ${\bar{x}}_{1}$ and ${\bar{x}}_{2}$ represent two different inputs: only ${\bar{w}}_{1}$ activates *Y*; only ${\bar{w}}_{2}$ activates *Y*; ${\bar{w}}_{1}$ and ${\bar{w}}_{2}$ are required to activate *Y*.

**Table 2.** Truth table with weights representing the presence of hyperedges in a continuous formulation for the graph shown in Figure 1

$x_{1}$	$x_{2}$	${\bar{B}}^{I}({\bar{x}}_{1},{\bar{x}}_{2})=...$
0	0	$0\cdot (1-{\bar{x}}_{1})\cdot (1-{\bar{x}}_{2})+$
0	1	$w_{1}\cdot (1-{\bar{x}}_{1})\cdot {\bar{x}}_{2}+$
1	0	$w_{2}\cdot {\bar{x}}_{1}\cdot (1-{\bar{x}}_{2})+$
1	1	$\text{OR}(w_{1},w_{2},w_{3})\cdot {\bar{x}}_{1}\cdot {\bar{x}}_{2}$

Note: The multivariate polynomial interpolation of the OR gate is used to make a smooth approximation of a logical disjunction for the weights *w*_1_, *w*_2_ and *w*_3_.

When solutions are of a binary nature this formulation holds exactly the same solution as the previously shown for the mixed integer nonlinear case. So far, this reformulation produces an over-parameterized problem which does meet the basic constraint that each hyperedge can only be present or not present. Thus, to enforce that solutions for *w* tend to be of a binary nature, we add a penalty. The objective function to be minimized becomes:

(6) $$\begin{array}{l} \underset{n,k,\tau ,w}{\text{minimize}}F_{p}=\underset{F}{\underset{︸}{\sum_{\epsilon =1}^{n_{\epsilon }}\sum_{o=1}^{n_{o}^{\epsilon }}\sum_{s=1}^{n_{s}^{\epsilon ,o}}{\left({\tilde{y}}_{s}^{\epsilon ,o}-y_{s}^{\epsilon ,o}\right)}^{2}}}+\underset{P}{\underset{︸}{\alpha \cdot \sum_{i=0}^{n_{int}}p_{w_{i}}}}\\ \text{subject to }\\ p_{w_{i}}=\{\begin{array}{cc} w_{i}, & \text{if }w_{i}\leq 0.5\\ 1-w_{i}, & \text{ if }w_{i}>0.5, \end{array}\\ 0\leq w\leq 1\\ {\text{LB}}_{n}\leq n\leq {\text{UB}}_{n}\\ {\text{LB}}_{k}\leq k\leq {\text{UB}}_{k}\\ {\text{LB}}_{\tau }\leq \tau \leq {\text{UB}}_{\tau }\\ \dot{\bar{x}}=f(\bar{x},n,k,\tau ,w,t)\\ \bar{x}(t_{0})={\bar{x}}_{0}\\ y=g(\bar{x},n,k,w,\tau ,t) \end{array}$$

where $p_{w_{i}}$ is the penalty associated with the deviation of each *w_i_* from the nearest binary value (0 or 1).

The usage of this relaxed formulation to find MIDO solutions can be summarized as follows:

- In a first phase, we solve the relaxed problem with a small or null penalty value to find a set of continuous parameters, which are able to describe the data well.
- The solution found in the previous iteration is used to restart eSS with a given *α*. Depending on the difficulty of the problem, this step might consist on only one iteration or multiple phases with increasing *α*.
- In a final step, we apply eSS to solve the pure MINLP problem, where the best solution from the previous steps is used as an initial guess (rounding the previously relaxed variables).

Here, *α* is chosen as a continuation parameter that gives a sequence of trade-offs between the penalty (*P*) and the squared residuals (*F*), with the final aim of getting *p_w_* (iteratively) close to zero. If *α* is increased too sharply, the penalty (*P*) will dominate over the goodness of fit (*F*) and we risk guiding the metaheuristic towards uninteresting areas of the search space.

The term goodness of fit refers to the quality of the adjustment of the model to the data and can be quantified using different metrics like the sum of the squared residuals (previously defined as *F*), the correlation coefficient between model predictions and experimental data or the root mean squared error (RMSE), defined as:

(7) $$\text{RMSE}=\sqrt{\frac{\sum_{\epsilon =1}^{n_{\epsilon }}\sum_{o=1}^{n_{o}^{\epsilon }}\sum_{s=1}^{n_{s}^{\epsilon ,o}}{\left({\tilde{y}}_{s}^{\epsilon ,o}-y_{s}^{\epsilon ,o}\right)}^{2}}{\sum_{\epsilon =1}^{n_{\epsilon }}\sum_{o=1}^{n_{o}^{\epsilon }}n_{s}^{\epsilon ,o}}}.$$

### 2.5 Remarks on the tuning and performance assessment of metaheuristics

Meta-heuristics for global optimization are approximate stochastic methods which in general do not have proofs of convergence. Thus it is not possible to obtain an analytical prediction of the effort it will take to arrive to a solution of a certain quality. Similarly, it is not possible to ensure that the metaheuristic will arrive to near-global solutions in every run. A related problem is the tuning of the internal search parameters of the method. Although the eSS metaheuristic is mostly self-adapting in that sense, we still need to choose a stopping criterion.

Due to this lack of theoretical guarantees and the stochastic behavior of these methods, one must resort to empirical tuning and performance assessments. We have performed this tuning and assessment based on repeated runs of the methods for each problem (see guidelines provided by Luke, 2013) and the subsequent analysis of the convergence curves (objective function values versus number of function evaluations) and the distributions of the solutions found (see general discussion in Chiarandini *et al.*, 2007).

The analysis of these distributions for a number of trial runs allow us to choose the stopping criteria. In general, stopping criteria for metaheuristics are based on 3 metrics (Glover and Kochenberger, 2003): (i) after a fixed number (budget) of function evaluations (FEs), computation time or iterations (ii) after a fixed number of iterations without improvement in the cost function (iii) when the cost function arrives to a pre-set value-to-reach.

These criteria can be combined. In our study, we have chosen (i) because criteria (ii) can be reached with premature stagnation in local optima, and criteria (iii) requires a priori knowledge about the global solution. Criteria (i) is widely used (Schoen, 2009) and is particularly useful when the evaluation of the cost function is computationally expensive (as in our study), since it also directly reflects practical limits on computation time.

## 3 Results

### 3.1 Case study 1: synthetic signaling pathway

In order to illustrate the methodology we now turn to a published model used by MacNamara *et al.* (2012). This dynamic model is composed by 26 ordinary differential equations and 86 continuous parameters. It was initially used to illustrate the capabilities and limitations of different formalisms related with logic-based models. Although this is a synthetic model, it was derived to be a plausible representation of a signaling transduction pathway. This model was used to generate pseudo-experimental data for 10 combinations of experimental perturbations of 2 ligands (TNF*α* and EGF) and two kinase inhibitors (for PI3K and RAF1). From a total of 26 dynamic states, 6 were observed (NFKB, P38, AP1, GSK3, RAF1 and ERK) and 5% of Gaussian noise was added to the data.

**Fig. 2.**
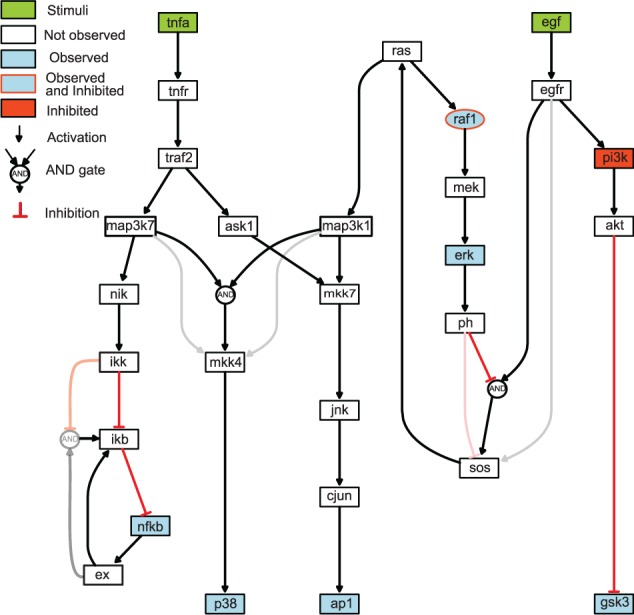
Case study 1 (synthetic signaling pathway): Hypergraph showing every possible logic gate consistent with the prior knowledge network. Strong red and dark hyperedges correspond to gates present in the original model used to generate the *in silico* data while grey and light red hyperedges show links not present in this model

Following the methodology described in Saez-Rodriguez *et al.* (2009), we obtained an expanded network containing every possible AND/OR logic gate given the initial graph structure. This so-called expansion procedure generated a nested model comprising 34 additional variables, one for each hyperedge (Fig. 2).

The model and experimental setup were implemented using AMIGO (Balsa-Canto and Banga, 2011) and method of choice for the simulation was CVODES (Serban and Hindmarsh, 2003).

As described previously, when using stochastic methods the recommended practice is to run each optimizer a number of times to assess their performance based on a distribution of results. This problem was solved in 30 runs by each method, ACOmi, MITS, eSS and MPeSS, using a budget of $6\cdot \;10^{4}$ function evaluations. In the case of MPeSS this budget was equally distributed among three phases, with the first two using relaxations with *α* = 1 and *α* = 3, and with the third solving the original MIDO problem.This parameter was chosen such that the penalty (*P*) is not generally dominating over squared residuals (*F*) (see Supplementary Fig. S3).

**Fig. 3.**
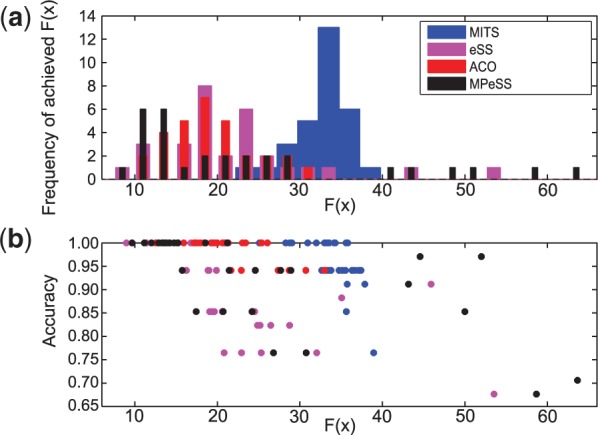
Case study 1 (synthetic signaling pathway): (**a**) Histogram of the final objective function achieved by each method (*F*(*x*)) across the multiple independent optimization runs. (**b**) The accuracy of the obtained solutions as a function of the objective function. Each dot describes the results of an independent optimization run

Albeit no solver/configuration was able to recover the correct solution in every run, the multi-phase strategy of MPeSS, was the most reliable method, i.e the method which located vicinity of the optimal solution more often. In Figure 3a, the histogram represents the distribution of final values achieved by each method. By combining both problem formulations (relaxed and MINLP), MPeSS is able to arrive to near-globally optimal values in approximately 47% of the runs. However, because MPeSS also has a large tail of poor solutions, the median of the final objective function values is similar to that of eSS and ACOmi. According to the non-parametric Wilcoxon rank-sum test, the three solvers did not show statistically significant differences (see Supplementary Table S2). MITS systematically failed to solve the problem for the considered FE budget. Convergence curves for the tested methods can be found in the Supplementary Materials (Supplementary Figs S2 and S3).

**Fig. 4.**
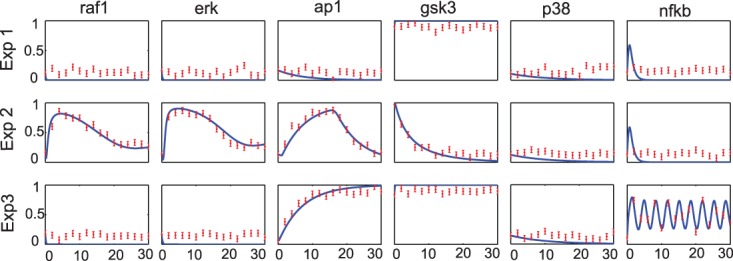
Case study 1 (synthetic signaling pathway): predicted versus observed time-series for the best solution found (experiments 1 and 2), showing a very good agreement of the simulation with the pseudo-experimental data used to calibrate the model

Figure 3b represents the accuracy of the obtained solution as a function of the final objective function value achieved. Each dot describes the result of an independent optimization run. Near-globally optimal solutions, with a final objective function value below a certain threshold, are always able recover the correct solution. The accuracy is computed as (TP+TN)/(TP+TN+FP+FN), where *TP* is the number of true positive, *TN* the number of true negative, *FP* the number of false positive and *FN* the number of false negative hyper edges when compared with the correct solution (an accuracy of 1). Since the data has been generated *in silico* with known structure (see Fig. 2) and parameters we can compute the accuracy of the recovered model structures. Additionally the time-course simulations (Fig. 4) indicate a very good agreement with the pseudo-experimental data, which is also indicated by its low RMSE of 0.099. A solution with poor goodness of fit (RMSE of 0.2659) is also given in the Supplementary Materials (Supplementary Fig. S9).

### 3.2 Case study 2: application to the KdpD/KdpE two-component signal transduction pathway

In this section, we consider a model of K^+^ regulation of the Kdpd/Kdpe two-component signal transduction pathway in *Escherichia coli*. The main components of this system are the high-affinity K^+^ transporter KdpFABC and two regulatory proteins, KdpD (sensor kinase) and KdpE (response regulator) (Laermann *et al.*, 2013). The two proteins regulate the kdpFABC operon, which is activated in response to K^+^ limiting conditions (Heermann and Jung, 2010), restoring the intracellular K^+^ concentration (Jung *et al.*, 2012).

Recently, new experimental data has been generated using mutant strains with impaired K^+^ properties and diverse K^+^ stimulation conditions. Based on these data, Rodriguez-Fernandez *et al.* (2013) have postulated the possible existence of two new possible feedback loops and an alternative expression for a previous description of the stimuli counteraction responsible for restoring K^+^ homeostasis. These new two feedback loops affected the translation and proteolysis of KdpFABC. Here, we write the differential equation describing the dynamics of KdpFABC as a logic-based ODE:

$$\frac{dKdpFABC}{dt}=$$

(8) $$\begin{array}{l} \left(w_{2}\cdot [1-f^{Hn}(\frac{mRNA}{norm_{mRNA}})]\cdot [1-f^{Hn}(KdpFABC)\right]\\ +0\cdot [1-f^{Hn}(\frac{mRNA}{norm_{mRNA}})]\cdot f^{Hn}(KdpFABC)\\ +OR(w_{1},w_{2},w_{3})\cdot f^{Hn}(\frac{mRNA}{norm_{mRNA}})\cdot [1-f^{Hn}(KdpFABC)]\\ +w_{1}\cdot f^{Hn}(\frac{mRNA}{norm_{mRNA}})\cdot f^{Hn}(KdpFABC)\\ -KdpFABC)\cdot \tau_{KdpFABC}, \end{array}$$

where *norm_mRNA_* is a parameter, used to scale mRNA to values between 0 and 1.

The expression for R3 controls the dephosphorylation of KdpEp:

(9) $$\frac{dR_{3}}{dt}=[w_{4}\cdot f^{Hn}(KdpFABC)-R_{3}]\cdot \tau_{R_{3}},$$

where it is assumed that an the increase in the KdpFABC transporter will decrease internal K^+^ concentration leading to an lower dephosporylation rate of KdpEp. The expanded model is composed by 4 hyperedges and 27 continuous parameters, mostly related with the original model by Rodriguez-Fernandez et al. (2013). More information about the model structure and context of this model can be found in the Supplementary Materials.

To evaluate the ability of our method to describe and calibrate a model in a realistic scenario where multiple hypothesis are postulated, we used the model derived by Rodriguez-Fernandez and colleagues to generate pseudo-experimental data. We considered 10 different scenarios by varying the external concentration of K^+^ and by considering a wild-type and a mutant strain. The mutant strain is modelled by removing the influence *R*_3_ in the dephosphorylation of KdpEp. In the 10 experimental scenarios KdpFABC and mRNA were observed and perturbed with 5% of Gaussian noise.

We executed 30 optimization runs for each solver, eSS, ACOmi and MITS using the objective function *F*. The same budget of objective function evaluations was given to every run. In this case due to the smaller size of the problem we did not see any improvement by using MPeSS over eSS. The most robust method was clearly eSS (see Supplementary Figs S11 and S12), a result which is supported by the non-parametric Wilcoxon rank-sum test (see Supplementary Table S3). ACOmi was also able to solve the problem in a few instances. MITS consistently failed to solve the problem for the allowed FE budget.

**Fig. 5.**
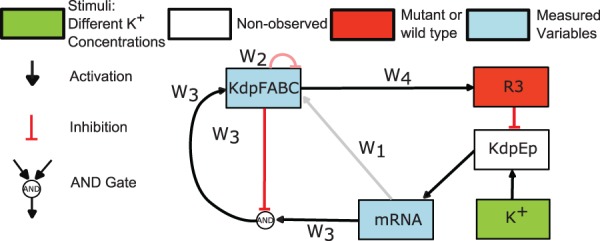
Case study 2 (*E.coli* homeostasis): The recovered model is depicted by strong red and dark hyperedges. Excluded hyperedges are represented in grey and light red

After redundant hypereges were filtered, all solutions showing a final objective function value below a given threshold (a total of 26) located the same solution. CellNOpt (Terfve *et al.*, 2012) was used to illustrate this solution (see Fig. 5). In this problem 4 binary variables were considered; *w*_1_, *w*_2_, *w*_3_ and *w*_4_. The hyperedges *w*_3_ and *w*_4_ were present in every of the top performing solutions while *w*_1_ and *w*_2_ were always absent.

When comparing the time course simulation of the best solution with the pseudo-experimental data we see that there is an excellent agreement between the two (normalized RMSE values of 0.0168 and 0.0191 for kdpFABC and mRNA, respectively).

### 3.3 Case study 3: signaling application to transformed liver hepatocytes

In this section, we explore the reverse engineering of a logic-based ODE model using liver cancer data (a subset of the data generated by Alexopoulos *et al.*, 2010). It consists of phosphorylation measurements from an hepatocellular carcinoma cell line (HepG2) at 0, 30 and 180 minutes after perturbation. Although the data-set covers only three sampling time points it includes a large combination of 64 perturbations comprising 7 ligands stimulating inflammation and proliferation pathways as well as 7 small-molecule inhibitors blocking the activity of key kinases (see Supplementary Fig. S17). Thus, despite having only 3 time points per measured variable, the rich experimental design ensures a large information content in the data, facilitating the reverse engineering process.

To preprocess the network, we used CellNOptR, the R version of CellNOpt (Terfve *et al.*, 2012). Basically, the network was compressed (see Supplementary Fig. S18) to remove as many non-observable/non-controllable species. Subsequently, it was expanded to generate all possible hyperedges (AND gates) formed by a pair of inputs. The obtained full network (Supplementary Fig. S19) has a total of 109 hyperedges and 135 continuous parameters. To transform this network into logic-based ODEsl, we developed a parser that generates a C file and Matlab scripts compatible with AMIGO (Balsa-Canto and Banga, 2011).

To use logic-based ODE models, all data should be in the [0,1] range and thus we simply normalized the data by rescaling it to this range. From the total 25 states present in the model, 16 corresponded to observed species. The initial conditions for the other 9 species are not known and were therefore estimated. In order not to increase the problem size and multi-modality unnecessarily estimated initials conditions were assumed the same for every of the 64-experiments.

The problem was solved in 20 independent instances by each solver: ACOmi, eSS and MPeSS. The first two methods used the *F* objective function, while the third method used the relaxed formulation objective function (*F_p_*). For this problem we considered a larger budget of 1.5·10^5^ FEs. The budget for MPeSS was split into 6 phases. The first 5 with increasing values of *α* and a final round configured as MINLP solver. As in case study 1 (the synthetic signaling pathway), *α* was chosen such that the penalty (*P*) was not generally dominating over the squared sum of the residuals (*F*) and increased gradually to facilitate convergence towards areas of the search space where the goodness of fit prevails.

MPeSS not only found the best solution but was also the most robust strategy (convergence curves are given in the Supplementary Fig. S20 for ACOmi/eSS and S.21 for MPeSS). This result is supported by the non-parametric Wilcoxon rank-sum test (see Supplementary Table S6). No significant differences were found between ACOmi and eSS which were occasionally able to find solutions with low objective function values (see histogram in Supplementary Fig. S22).

**Fig. 6.**
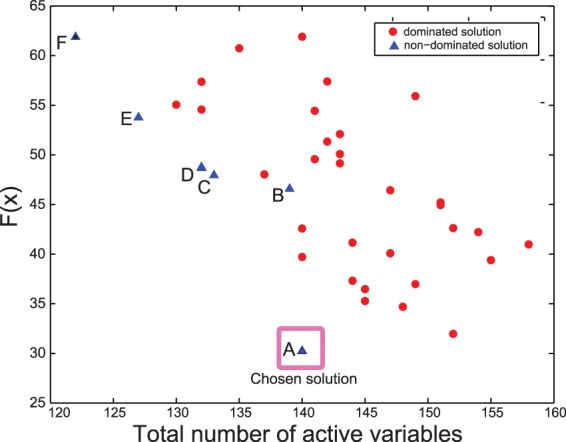
Case study 3 (HepG2): This figure shows the Pareto front for the trade-off between the goodness of fit (*F* ) obtained by each independent optimization run and the number of active variables (number of active binary variables plus the number of active continuous parameters), which is a proxy for model complexity. The chosen solution shows a good balance between goodness of fit (RMSE of 0.121) and complexity

In Figure 6 we show, for the best solutions (cost function under 65) the goodness of fit (*F*) obtained by each independent optimization run as a function of the number of active variables, i.e*.* the number of binary variables plus the number of continuous parameters. Here we considered solutions in which the final objective function value is up to two times worse than best found. In general, one applies Occam’s razor, i.e*.* we seek the simplest model which can explain the available data satisfactorily. The best model structure (solution A) achieved a RMSE of 0.1211. Comparing with other solutions, it shows a good balance between goodness of fit (*F*) and complexity (see Fig. 6). Model structures for solutions A–F (Supplementary Figs S27–S32) along with goodness of fit measures (Supplementary Fig. S26) are given in the Supplementary Materials.

Despite the uncertainty in the completeness of the PKN and the uncertainty in the experimental data, we are able to find relatively simple mechanistic models which explain the data. The agreement between the simulation and the experimental data is qualitatively and quantitatively good with the transient behaviour of phosphorylated proteins being well captured by the dynamic model depending on the different stimuli and inhibitors (trajectories available in the Supplementary Figs S33–S36).

## 4 Conclusion

In this contribution, we apply a mixed-integer global optimization approach to reverse engineer logic-based ODE models from time-course data. The problem is stated as simultaneously finding the binary variables that determine the model structure and its associated continuous parameters. Further, to improve computational efficiency, we present a relaxed non-linear programming reformulation of the problem that allows us to find good initial points for the mixed-integer nonlinear programming problem.

With our approach, we are able to find a number of solutions which describe the data satisfactorily. It is important to highlight that the lack of unique solutions is common in reverse engineering problems. Even in the utopian case of large amounts of perfect data available, the reverse engineering of dynamic models can have non-unique solutions, and this is independent of the method used to recover them. For example, in the case of chemical reaction networks it has been shown that many network configurations can describe the same dynamical behavior (Szederkényi *et al.*, 2011).

Although the metaheuristic approach we present does not provide guarantees about the global optimality of the solutions, we show, by solving synthetic problems (case studies 1 and 2), that problems of realistic size can be successfully solved with a reasonable effort.

In the third case study, we apply the methods to a large signaling network given real experimental data from a liver cancer cell line (HepG2). Due to its size (109 binary variables and 135 continuous parameters) this is, from the optimization point of view, an extremely challenging problem and illustrates well the capability of the method regarding problems of realistic size. Here we did not recover unique solutions, as was expected due to the lack of structural identifiability typical of these problems: their underdetermined nature (Siegenthaler and Gunawan, 2014)and the corresponding indistinguishability and non-uniqueness (Szederkényi *et al.*, 2011). Instead, we did find a family of solutions much simpler than the original superstructure containing all likely interactions, with a very good fit to the experimental data. This is illustrated in the Supplementary Materials by the initial expanded superstructure (Supplementary Fig. S19) and the family of obtained solutions (Supplementary Fig. S24). This family of solutions has the potential to be exploited by approaches like ensemble modeling (Kuepfer *et al.*, 2007).

Although the obtained results are very encouraging, future work will focus on further improving the efficiency of the metaheuristic optimization methods by exploiting multi-method cooperation and high-performance computing (parallelization).

## Supplementary Material

Supplementary Data
