# Supplementary material for: Reverse engineering of logic-based differential equation models using a mixed-integer dynamic optimization approach
Source: Bioinformatics. 2015 May 21;31(18):2999–3007. doi: 10.1093/bioinformatics/btv314 (PMC4565031; doi:10.1093/bioinformatics/btv314)
Supplement: Supplementary Data [file supp_31_18_2999__index.html]

Reverse engineering of logic-based differential equation models using a mixed-integer dynamic optimization approach — Reverse engineering of logic-based differential equation models using a mixed-integer dynamic optimization approach — Supplementary Data 

# Reverse engineering of logic-based differential equation models using a mixed-integer dynamic optimization approach

## Supplementary Data

files

- Supplementary Data - pdf file
