# Supplementary material for: Reverse engineering of logic-based differential equation models using a mixed-integer dynamic optimization approach
Source: Bioinformatics. 2015 May 21;31(18):2999–3007. doi: 10.1093/bioinformatics/btv314 (PMC4565031; doi:10.1093/bioinformatics/btv314)
Supplement: Supplementary Data [file supp_btv314_supplementary_BIOINF-2014-1450.R3.pdf]

Supplementary Materials

Reverse engineering of logic-based models using a mixed-integer dynamic optimization approach

David Henriques, Miguel Rocha, Julio Saez-Rodriquez, Julio R. Banga

April 9, 2015

Contents

1 Case study 1: A synthetic signaling pathway model 5

2 Case Study 2: Application to the KdpD/KdpE two-component signal transduction pathway 16

3 Case Study 3: Signaling application to transformed liver hepatocytes (HepG2) 26

4 A brief comparison with the two time points Boolean approach 52

4.1 The synthetic pathway model 52

4.2 The HepG2 model 55

List of Figures

S.1 Case study 1 (synthetic signaling pathway): Expanded synthetic pathway model. 7

S.2 Case study 1 (synthetic signaling pathway): Convergence curves for MINLP solutions the synthetic pathway model. 8

S.3 Case study 1 (synthetic signaling pathway): Convergence curves for the relaxed MINLP solutions the synthetic pathway model. . 9

S.4 Case study 1 (synthetic signaling pathway): Summary of results obtained with each optimization method while solving the synthetic pathway problem. 10

S.5 Case study 1 (synthetic signaling pathway): Boxplot of the final objective function values for each optimization method (synthetic pathway problem). 11

S.6 Case study 1 (synthetic signaling pathway): Frequency of hyperedges in the synthetic pathway model for near optimal solutions with objective function values under 20. 12

S.7 Case study 1 (synthetic signaling pathway): Frequency of hyperedges in the synthetic pathway model for near optimal solutions with objective function values under 15 13

S.8 Case study 1 (synthetic signaling pathway): Predicted versus observed time-series for the best solution found. 14

S.9 Case study 1 (synthetic signaling pathway): Predicted versus observed time-series for the worst solution found. 15

S.10 Case study 2 (*E. coli* homeostasis): Convergence curves for the logic-based implementation of the KdpD/KdpE two-component system problem. 20

S.11 Case study 2 (*E. coli* homeostasis): Histogram of achieved objective function values and accuracy of solution for logic-based implementation of the KdpD/KdpE two-component system problem 21

S.12 Case study 2 (*E. coli* homeostasis): Boxplot of the final objective function value for each optimization method (eSS, ACO, MITS) while solving the KdpD/KdpE two-component system problem. . 22
S.13 Case study 2 (*E. coli* homeostasis): Predicted versus observed time series for the best solution found 23
S.14 Case study 2 (*E. coli* homeostasis): Frequency of hyperedges in the model for near optimal solutions with objective function values under 1500. 24
S.15 Case study 2 (*E. coli* homeostasis): Frequency of hyperedges in the model for near optimal solutions with objective function values under 1000. 25
S.16 Case study 3 (HepG2): Experimental data for the HepG2 cell-line case study. 28
S.17 Case study 3 (HepG2): original PKN before being compressed . 31
S.18 Case study 3 (HepG2): PKN network after compression. 32
S.19 Case study 3 (HepG2): Hypergraph after expansion. 33
S.20 Case study 3 (HepG2): Convergence curves for the HepG2 cell-line problem. 34
S.21 Case study 3 (HepG2): Convergence curves using MPeSS. 35
S.22 Case study 3 (HepG2): Histogram of the final objective function value achieved by each optimizer. 36
S.23 Case study 3 (HepG2): Boxplot of the final objective function value achieved by each optimizer (MPeSS, eSS, ACO). 37
S.24 Case study 3 (HepG2): Frequency of hyperedges in the model for near optimal solutions with objective function values under 37 . 38
S.25 Case study 3 (HepG2): Pareto front of the obtained solutions, showing the trade-off between complexity and fit 39
S.26 Case study 3 (HepG2): Experimental versus predicted data points for each of the non-dominated solution. 40
S.27 Case study 3 (HepG2): Network structure for solution A (best trade-off) 41
S.28 Case study 3 (HepG2): Network structure for solution B 42
S.29 Case study 3 (HepG2): Network structure for solution C 43
S.30 Case study 3 (HepG2): Network structure for solution D 44
S.31 Case study 3 (HepG2): Network structure for solution E 45
S.32 Case study 3 (HepG2): Network structure for solution F 46
S.33 Case study 3 (HepG2): Data and model predictions for solution A (experiments 1 to 16). 47
S.34 Case study 3 (HepG2): Data and model predictions for solution A (experiments 17 to 32) 48
S.35 Case study 3 (HepG2): Data and model predictions for solution A (experiments 33 to 48) 49
S.36 Case study 3 (HepG2): Data and model predictions for solution A (experiments 49 to 64) 50
S.37 The Boolean model found by the two time points approach in the synthetic pathway case study 53

S.38 Fitness of the best Boolean model found in the synthetic pathway case study . . . 54
S.39 The Boolean model found by the two time points approach in the HepG2 case study . . . 57
S.40 Fit of the best Boolean model found in the HepG2 case study (observables 1 to 8) . . . 58
S.41 Fit of the best Boolean model found in the HepG2 case study (observables 9 to 16) . . . 59

List of Tables

S.1 Case study 1, synthetic signaling pathway: Experimental setup for the synthetic pathway problem. . . . 6
S.2 Case study 1 (synthetic signaling pathway): The Wilcoxon rank sum test applied to the distributions of final objective function values obtained with each pair of methods. . . . 16
S.3 Case study 2 (*E. coli* homeostasis): The Wilcoxon rank sum test applied to the distributions of final objective function values obtained with each pair of methods. . . . 26
S.4 Case study 3 (HepG2): Stimuli and inhibitor values from experiments 1 to 32. . . . 29
S.5 Case study 3 (HepG2): Stimuli and inhibitor values for experiments 33 to 64. . . . 30
S.6 Case study 3 (HepG2): The Wilcoxon rank sum test applied to the distributions of final objective function values obtained with each pair of methods. . . . 51

1 Case study 1: A synthetic signaling pathway model

In this section we provide detailed results, including optimization statistics, obtained while solving case study 1. This case study is based on a synthetic signaling pathway model developed by [6]. The continuous parameters used to generate the pseudo-experimental data can be found in the supplementary materials of [6].

This section contains the following elements (in this order):

1. Experimental setup for the synthetic pathway problem.
2. Expanded synthetic pathway model.
3. Convergence curves for MINLP solutions.
4. Convergence curves for the relaxed MINLP solutions.
5. Summary of results obtained with each optimization method.
6. Boxplot of the final objective function value for each optimization method.
7. Frequency of hyperedges in the synthetic pathway model for near optimal solutions with objective function values under 20.0.
8. Frequency of hyperedges in the synthetic pathway model for near optimal solutions with objective function values under 15.0.
9. Experimental versus predicted time-series for the best solution found for synthetic pathway problem.
10. Experimental versus predicted time-series for the best solution found for synthetic pathway problem.
11. The Wilcoxon rank sum test for equal medians applied to the obtained distributions of final objective function values.

Experiment	IFNG	TNFA	IL1Ainh	IL6inh
1	0	0	0	0
2	1	0	0	0
3	0	1	0	0
4	1	1	0	0
5	1	0	1	0
6	0	1	1	0
7	1	1	1	0
8	1	0	0	1
9	0	1	0	1
10	1	1	0	1

Table S.1: *Case study 1, synthetic signaling pathway: Experimental setup for the synthetic pathway problem.* IFNG and TNFA are cytokines and can be regarded as an upstream model stimuli which triggers the signaling cascade. IL1Ainh and IL16inh are small-molecule inhibitors for IL1A and IL6.

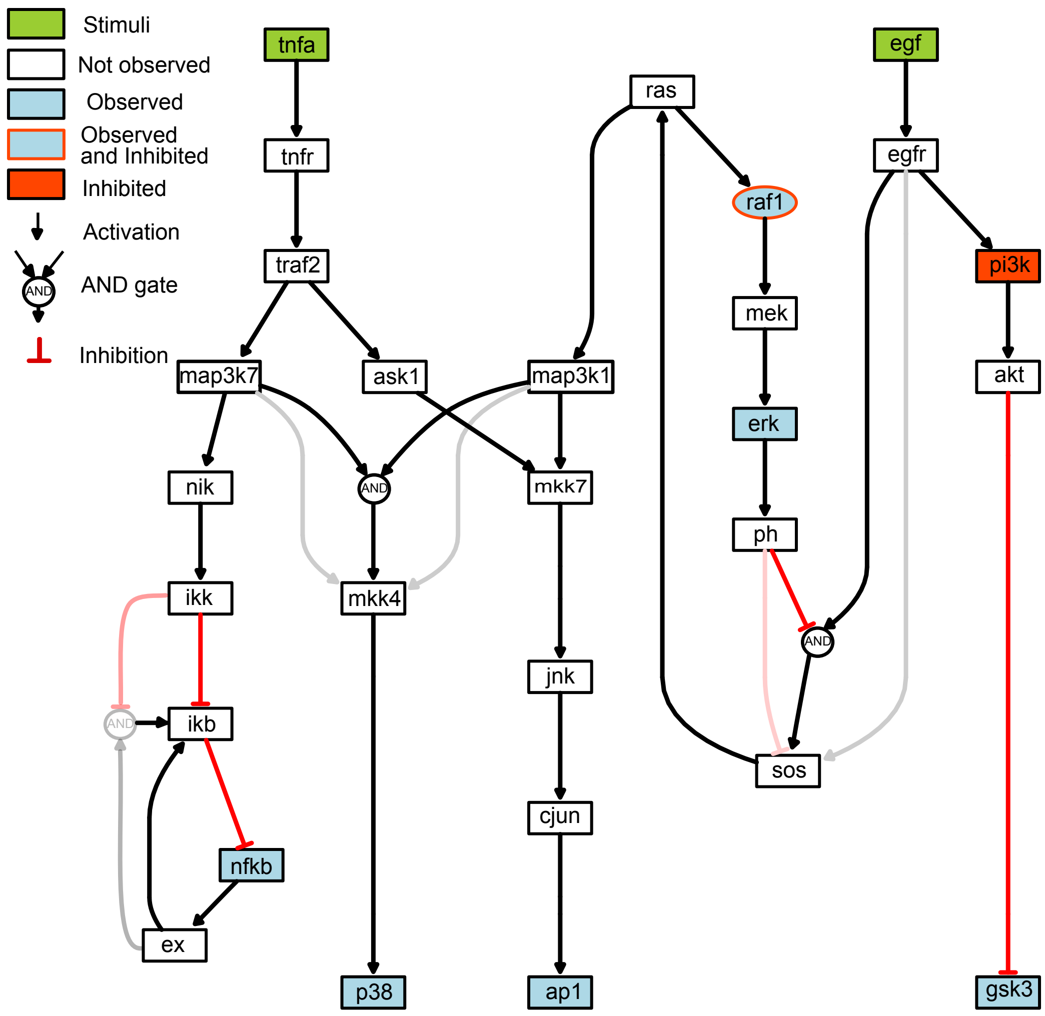

Figure S.1: *Case study 1 (synthetic signaling pathway): Expanded synthetic pathway model.* The original protein signaling network is expanded into a Boolean model containing every possible boolean gate in the form of hyperdges. The model contains 26 dynamic states, 86 continuous parameters and 34 possible hyperedges.

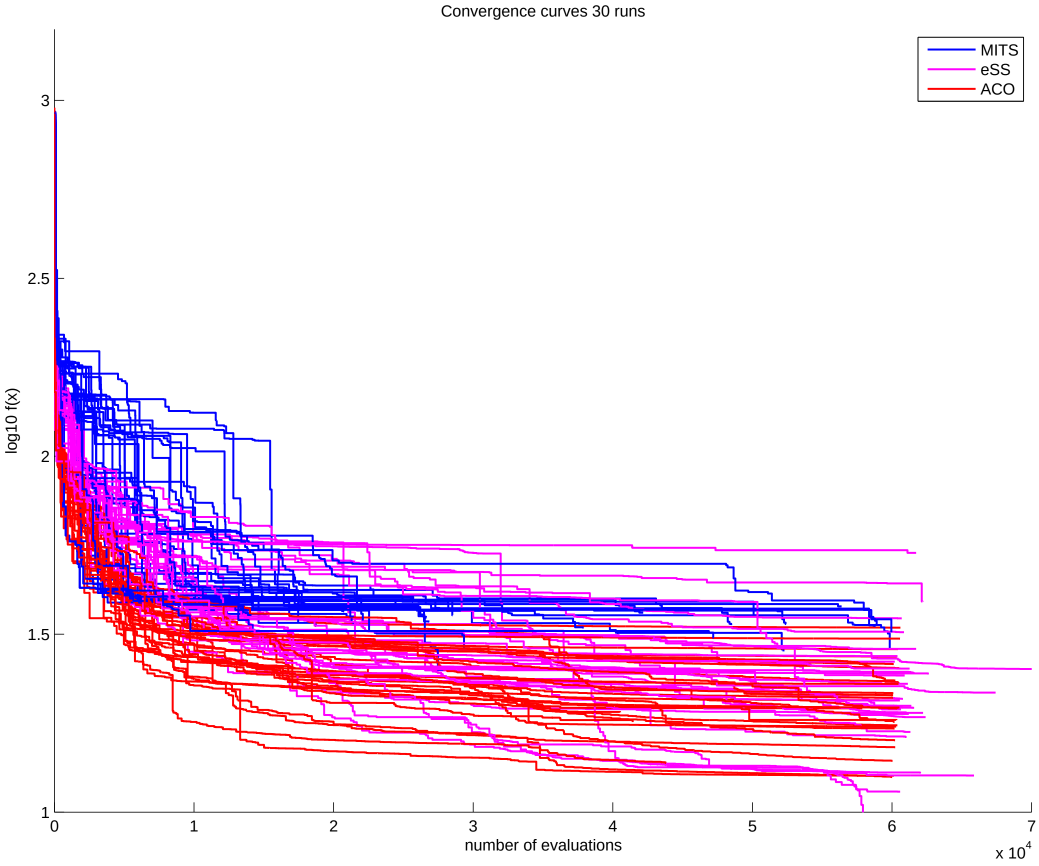

Figure S.2: *Case study 1 (synthetic signaling pathway): Convergence curves for MINLP solutions the synthetic pathway model.* The MINLP problem was solved using 30 independent runs for each solver (eSS, ACOmi and MITS). No method was always able to solve the problem for the given function evaluation budget. Although eSS found the best solutions, its overall performance was comparable to that of ACOmi. MITS was unable to solve the problem.

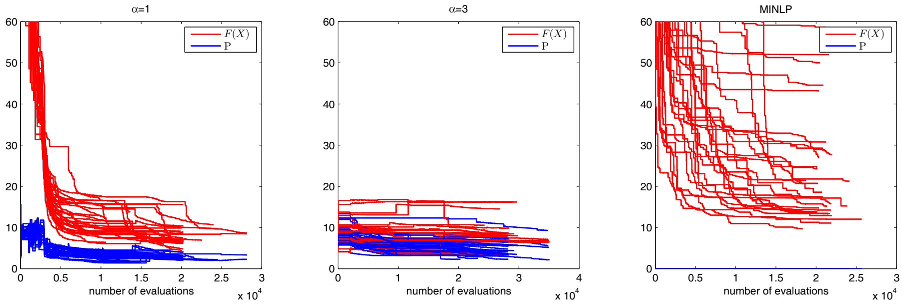

Figure S.3: *Case study 1 (synthetic signaling pathway): Convergence curves for the MPeSS solutions the synthetic pathway model.* The optimization procedure was divided into three phases. First, we solved the relaxed problem using eSS with α equal to 1. Next we used a family of solution found by eSS in the first step to feed another relaxed NLP but this time penalizing the deviation of w_i from 0 or 1 with α equal to 3. Finally we used the solutions from step 2 to start an MINLP problem solved by eSS.

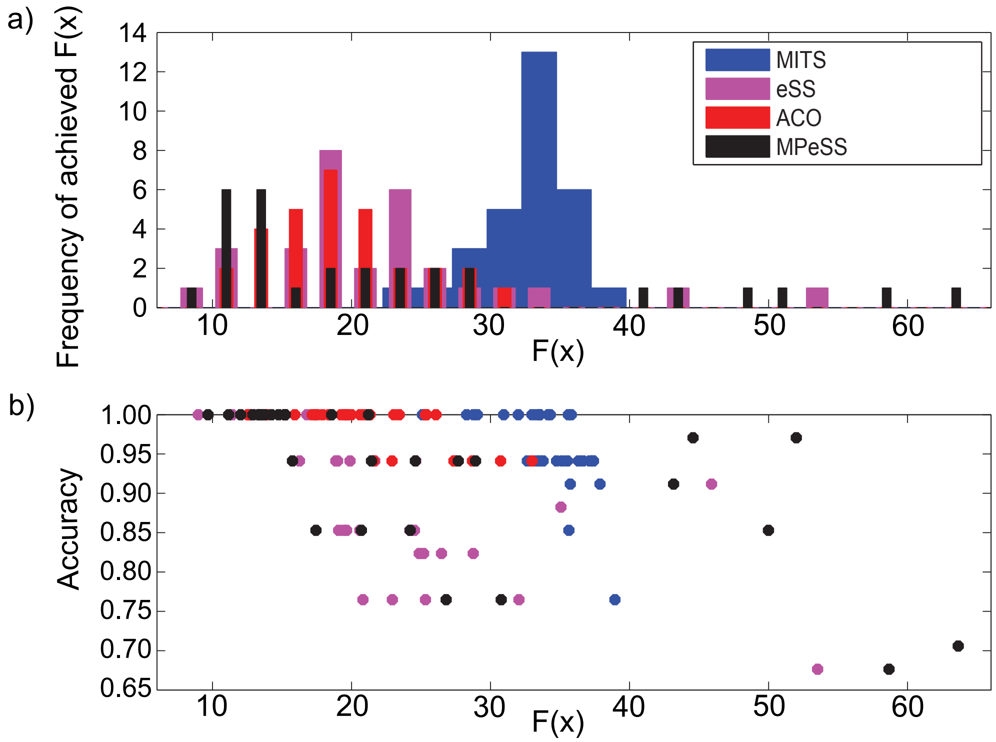

Figure S.4: *Case study 1 (synthetic signaling pathway): Summary of results obtained with each optimization method while solving the synthetic pathway problem.* The upper plot shows the histogram of the best objective function value achieved with each solver. The problem was solved using 30 independent optimization runs for each solver (eSS, MPeSS, ACOmi and MITS). The upper plot shows the frequency of the final objective function achieved. The lower figure shows the accuracy of the obtained solution computed as $(TP + TN)/(TP + TN + FP + FN)$ where TP is the number of true positive, TN the number of true negative, FP the number of false positive and FN the number of false negative hyperedges when compared with the correct solution. MPeSS was the best performer. Although eSS did slightly better, both eSS and ACOmi are competitive. MITS was systematically unable to solve the problem. Results show that for objective function values below a certain threshold, we can recover the correct model structure.

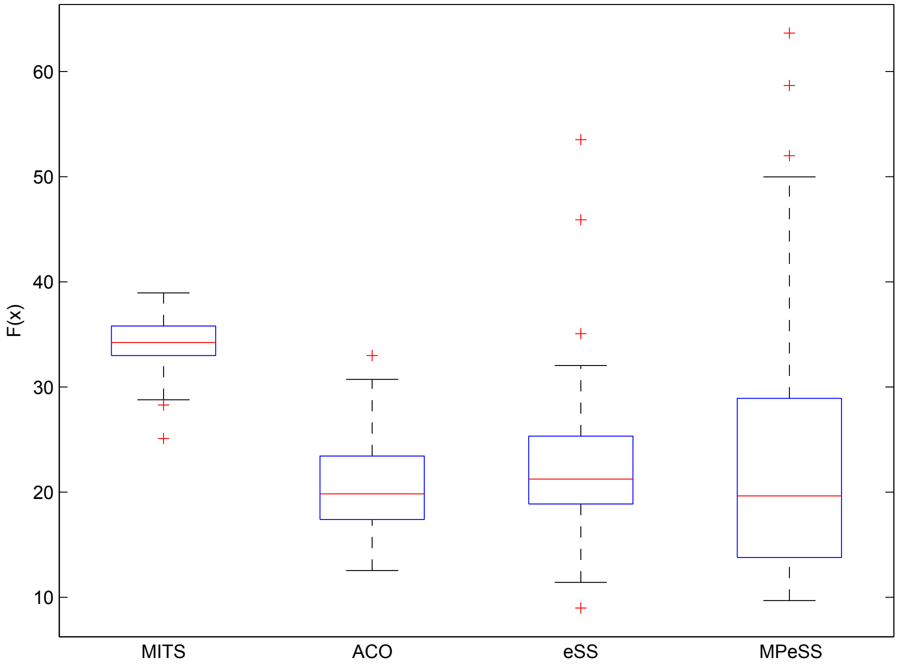

Figure S.5: *Case study 1 (synthetic signaling pathway): Boxplot of the final objective function value for each optimization method (synthetic pathway problem).*

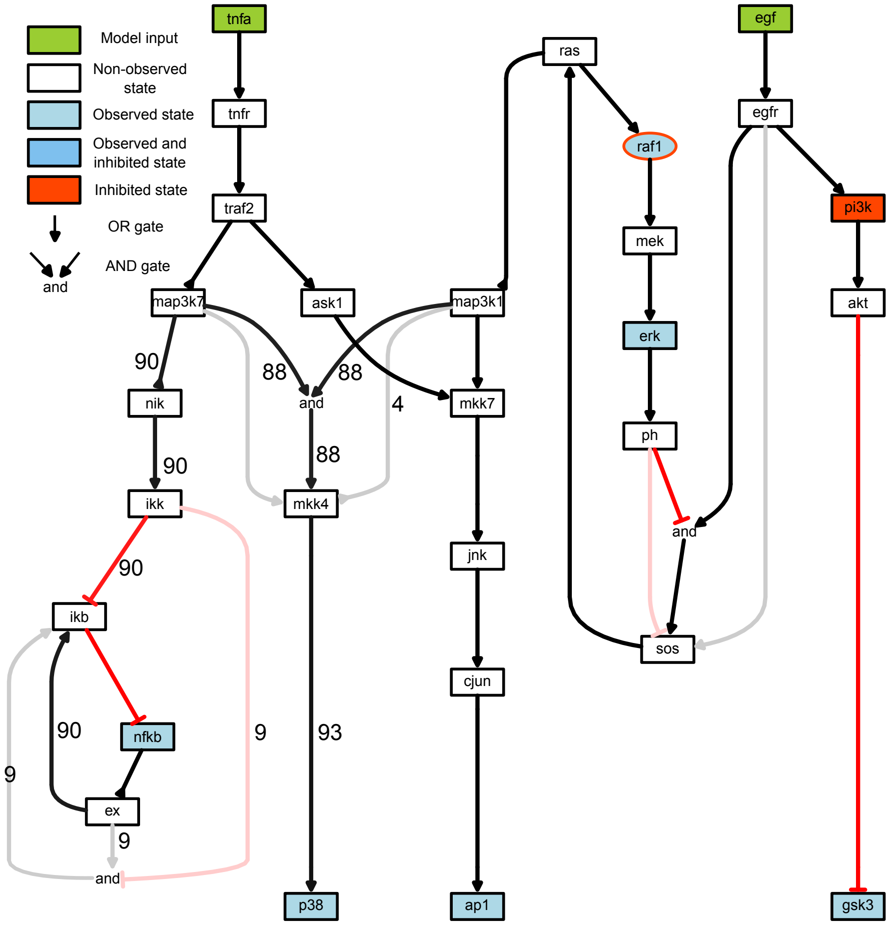

Figure S.6: *Case study 1 (synthetic signaling pathway): Frequency of hyperedges in the model for near optimal solutions with objective function values under 20.* The CellNOptR [8] software was used to illustrate specific hyperedges appear in near optimal solutions. In this example we consider solutions with an objective function value below 20. Redundant hypereges were filtered after the optimization procedure. The numbers stand for the percentage each hyperedge appeared in the final solutions. Strong dark or red links without any numbering illustrate links that are always present. Light gray or red line corresponds to hyperedges which are not present or are present in only a fraction of near optimal solutions below specified threshold.

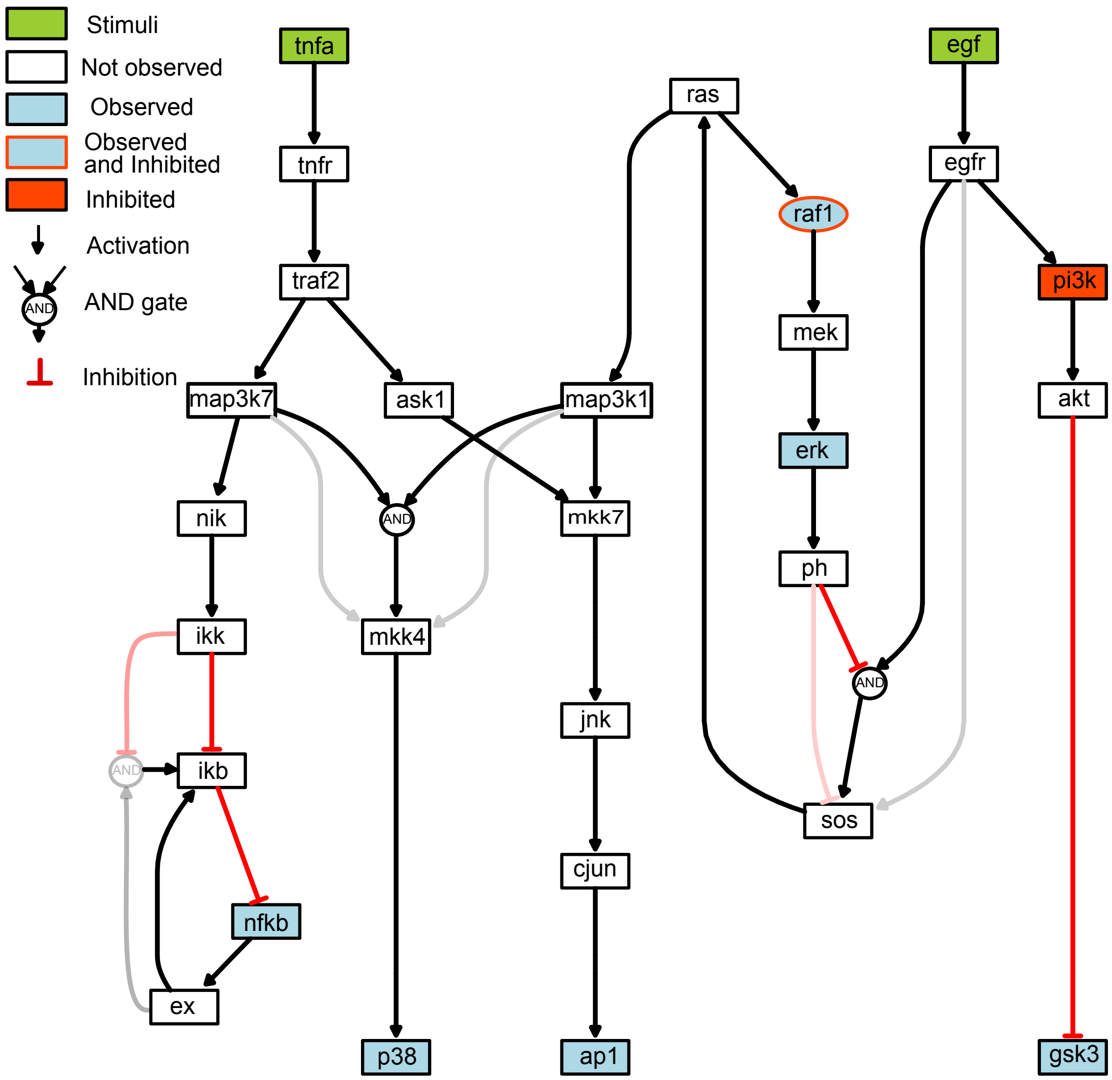

Figure S.7: *Case study 1, synthetic signaling pathway:Frequency of hyperedges in the model for near optimal solutions with objective function values under 15.* The CellNOptR [8] software was used to illustrate specific hyperedges appear in near optimal solutions. In this example we consider solutions with an objective function value bellow 15. Redundant hypereges were filtered after the optimization procedure. The numbers stand for the percentage each hyperedge appear in the final solutions. Strong dark or red links without any numbering illustrate links that are always present.

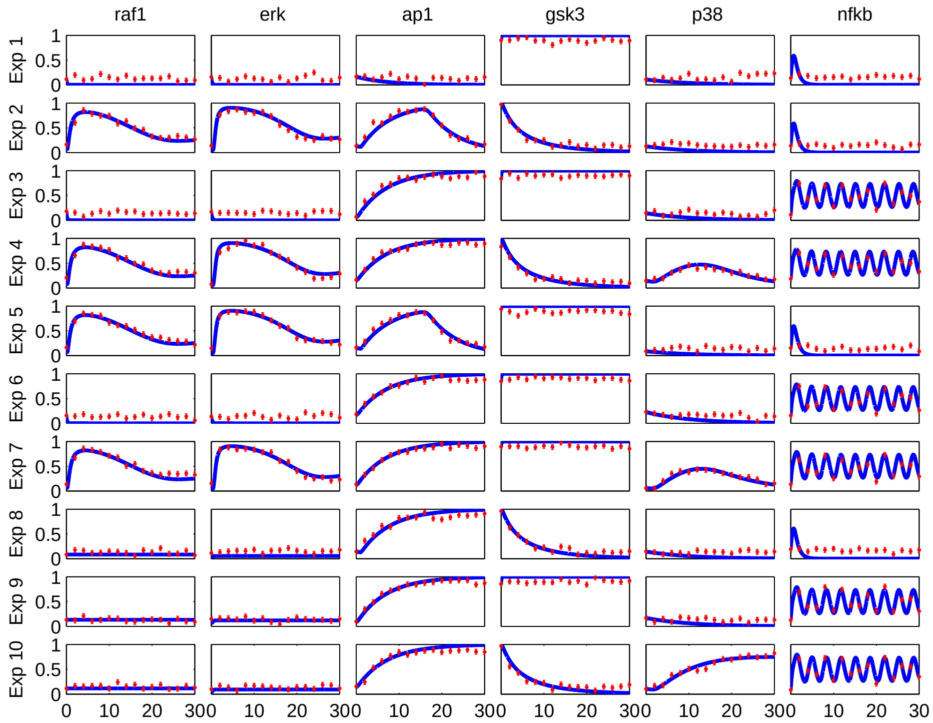

Figure S.8: *Case study 1 (synthetic signaling pathway): Predicted versus observed time-series for the best solution found.*

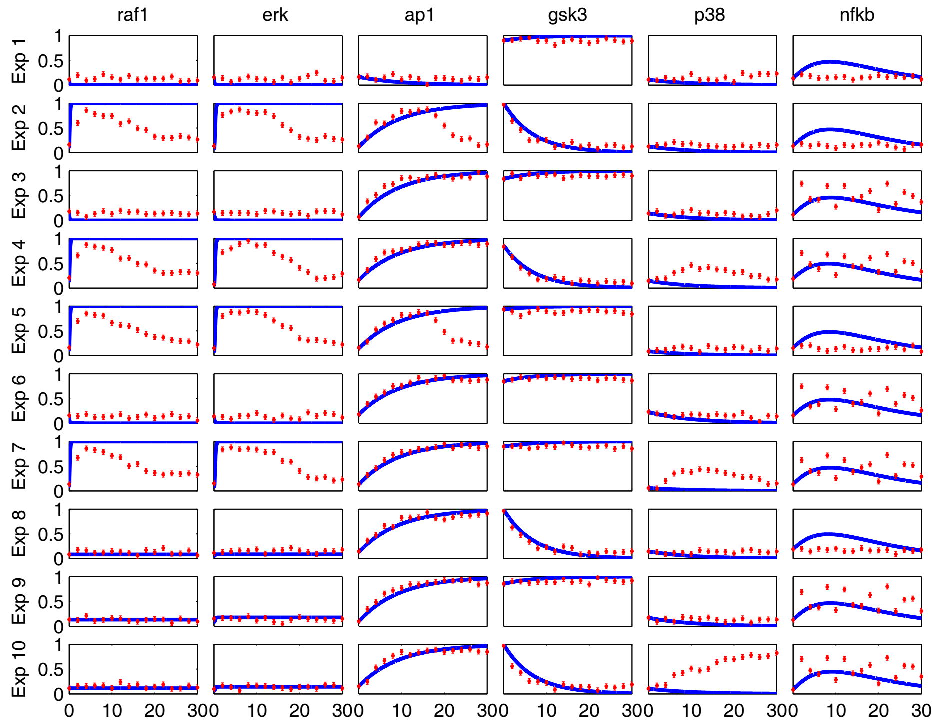

Figure S.9: *Case study 1 (synthetic signaling pathway): Predicted versus observed time-series for the worst solution found.*

	MITS(34.2)	eSS(21.2)	ACO(19.8)	MPeSS(19.6)
MITS(34.2)	–	$H = 1, P = 9.83 \cdot 10^{-8}$	$H = 1, P = 1.61 \cdot 10^{-10}$	$H = 1, P = 1.17 \cdot 10^{-4}$
eSS(21.2)	$H = 1, P = 9.83 \cdot 10^{-8}$	–	$H = 0, P = 0.348$	$H = 0, P = 0.579$
ACO(19.8)	$H = 1, P = 1.61 \cdot 10^{-10}$	$H = 0, P = 0.348$	–	$H = 0, P = 0.912$
MPeSS(19.6)	$H = 1, P = 1.17 \cdot 10^{-4}$	$H = 0, P = 0.579$	$H = 0, P = 0.912$	–

Table S.2: *Case study 1 (synthetic signaling pathway): The Wilcoxon rank sum test applied to the distributions of final objective function values obtained with each pair of methods.* The null hypothesis (H=0) is that both distributions have the same median (under parentheses) with a given probability (P).

2 Case Study 2: Application to the KdpD/KdpE two-component signal transduction pathway

In this section we provide further details and results regarding case study 2:

1. Further details about the model and the calibration case study considered.
2. Convergence curves using 3 solvers (eSS, MITS, ACO).
3. Histogram of final objective function values and their accuracy using 3 solvers (eSS, MITS, ACO).
4. Boxplot of the final objective function value for each optimization method (eSS, MITS, ACO)
5. Experimental versus predicted time series for the best solution.
6. Frequency of hyperedges in the model for near optimal solutions with objective function values under 1500.
7. Frequency of hyperedges in the model for near optimal solutions with objective function values under 1000.
8. The Wilcoxon rank sum test for equal medians applied to the obtained distributions of final objective function values.

In broad terms we used the model and experimental design described in [7] to generate a synthetic problem. We have modified this model to incorporate logic-based ODE expressions. This procedure is described in the text below:

Here we apply the methods to perform model selection in a model of K+ *E. coli* regulation of the KdpD/KdpE two-component signal transduction pathway. The main components of this system are the high-affinity K^+ transporter KdpFABC and two regulatory proteins KdpD (sensor kinase) and KdpE (response regulator) [5]. The two proteins regulate the kdpFABC operon, which is activated in response to K^+ limiting conditions [1], restoring the intracellular K^+ concentration [2].

[4] developed a first version of a differential algebraic equation model for this system. This model assumes that the stimuli enters the system through the dephosphorylation of the response regulator KdpE. Since the model did not include a functional transporter for K^+, to integrate stimuli counteraction of low K^+ levels by the KdpFABC K^+ uptake system, the authors introduced an empirical black box mediating the dephosphorylation of the response regulator (KdpE) depending on the K^+ concentration. Based in *in vitro* and *in vivo* measurements, Kremmling and colleagues were able to calibrate their model in order to reproduce the available data accurately.

Recently, new experimental data aiming further insight into the KdpE/KdpE two-component system has been generated. These experiments were based on mutant strains with impaired K^+ properties and diverse K^+ stimulation conditions. Based in these data, [7] and colleagues have postulated the possible existence of two new possible feedback loops ($R1$ and $R2$) and an alternative expression for the previously existing empirical black-box describing stimuli counteraction ($R3$). These new two feedback loops affected the translation ($R1$) and proteolysis ($R2$) of KdpFABC:

$$\frac{dKdpFABC}{dt} = R1 \cdot k_{tl3} \cdot mRNA - (k_{d2} \cdot R2 + \mu) \cdot KdpFABC \qquad (1)$$

where $R1$ and $R2$ are Hill shaped functions, k_{tl3} is the rate constant for translation, μ the growth rate and k_{d2} the degradation constant for KdpFABC. To calibrate this model a simultaneous model discrimination and parameter estimation process was done relying to the Akaike criterion, an objective function which promotes model parsimony.

In this work we adapted the model developed in [7] to the C language and solved it using CVODES. The original matlab version of this model contained 6 ODEs and a differential algebraic equation (DAE). Since CVODES does not handle DAEs we implemented an equivalent ODE and verified that both models produced the same results. Moreover, in order to consider the logic-based ODE framework we have rewritten the differential equation describing the dynamics of KdpFABC as follows:

$$\frac{dKdpFABC}{dt} = \tag{2}$$

$$\Bigg(0\cdot \Big[1 - f^{Hn}\Big(\frac{mRNA}{norm_{mRNA}}\Big)\Big]\cdot \Big[1 - f^{Hn}(FABC)\Big]$$

$$+ w_2\cdot \Big[1 - f^{Hn}\Big(\frac{mRNA}{norm_{mRNA}}\Big)\Big]\cdot f^{Hn}(KdpFABC)$$

$$+ OR(w_1, w_2, w_3)\cdot f^{Hn}\Big(\frac{mRNA}{norm_{mRNA}}\Big)\cdot \Big[1 - f^{Hn}(KdpFABC)\Big]+$$

$$+ w_1\cdot f^{Hn}\Big(\frac{mRNA}{norm_{mRNA}}\Big)]\cdot f^{Hn}(KdpFABC)$$

$$- KdpFABC\Bigg)\cdot \tau_{KdpFABC} \tag{3}$$

The expression for R3 controls the dephosphorylation of KdpE. Rewriting KdpE as a logic based ODE would require a major transformation of the model. To avoid this, for the stimuli counteraction we opted to add an additional dynamical state ($R3$) instead of using just a black box function:

$$\frac{dR_3}{dt} = \big[w_4\cdot f^{Hn}(KdpFABC) - R_3\big]\cdot \tau_{R_3} \tag{4}$$

After these transformations the model considered in this work had a total of 8 dynamical states.

To evaluate the ability of our method to describe and calibrate a model in a realistic scenario where multiple hypothesis are postulated, we used the model derived by Rodriguez-Fernandez and colleagues to generate pseudo-experimental data. We considered 10 different scenarios by varying the external concentration of K^+ and by considering a wild-type and a mutant strain. The mutant strain is modelled by removing the influence R_3 in the dephosphorylation of KdpEp. In the 10 experimental scenarios only KdpFABC and mRNA were observed and were added 5% of Gaussian noise.

We executed 30 optimization runs for each solver, eSS, ACOmi and MITS. The same budget of objective function evaluations was given to every run. In this case due to the smaller size of the problem we did not see any improvement by using MPeSS over eSS. The most robust method was clearly eSS (see Figures S.9 and S.10 in the supplementary materials). ACOmi was also able to solve the problem in a few instances. MITS consistently failed to solve the problem for the allowed FE budget.

After redundant hypereges were filtered, all solutions showing a final objective function value below a given threshold (a total of 26) located the same solution. The CellNOptR [8] software was used to illustrate this solution (see Figure S.15). In this problem 4 binary variables were considered; w_1, w_2 , w_3 and w_4. The hyperedges w_3 and w_4 were present in every of the top performing solutions while w_1 and w_2 were always absent.

When comparing the time course simulation of the best solution with the pseudo-experimental data we see that there is an excellent agreement between the two (normalized RMSE values of 0.0168 and 0.0191 for kdpFABC and mRNA, respectively).

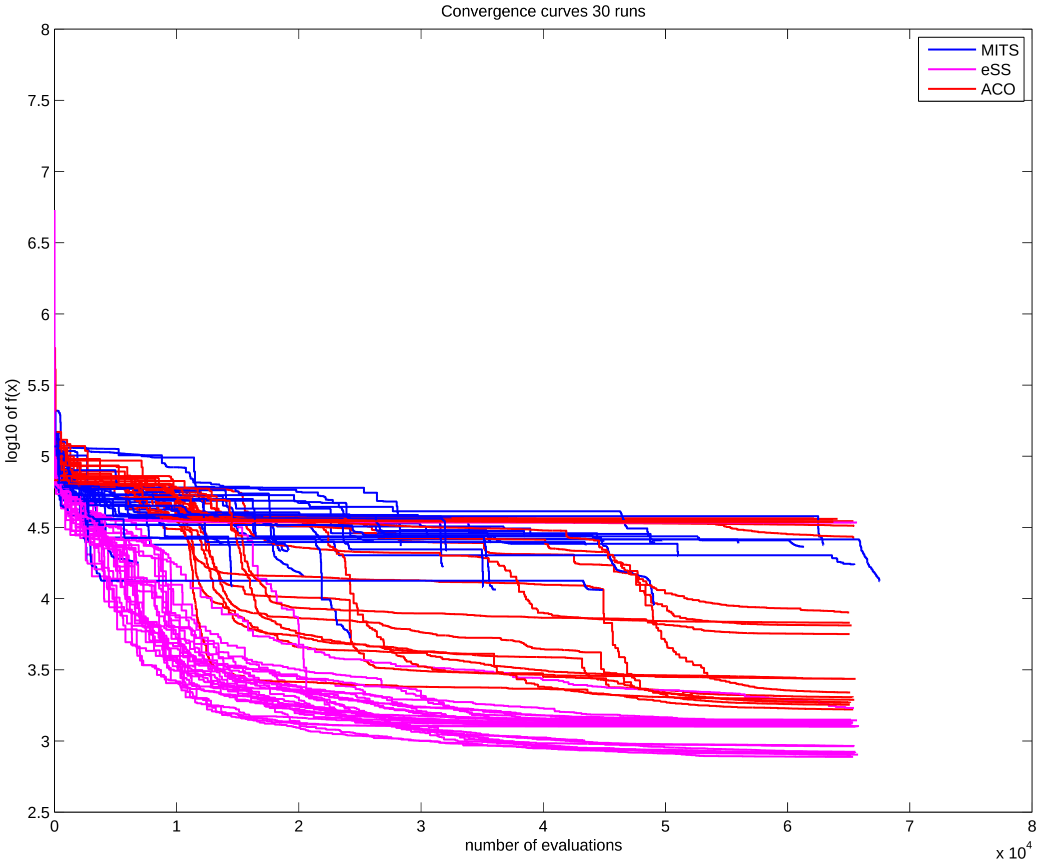

Figure S.10: *Case study 2 (E. coli homeostasis): Convergence curves for the logic-based implementation of the KdpD/KdpE two-component system problem.* The convergence curves show that eSS is the best performer, finding near globally optimal solutions in the allowed function evaluation budget.

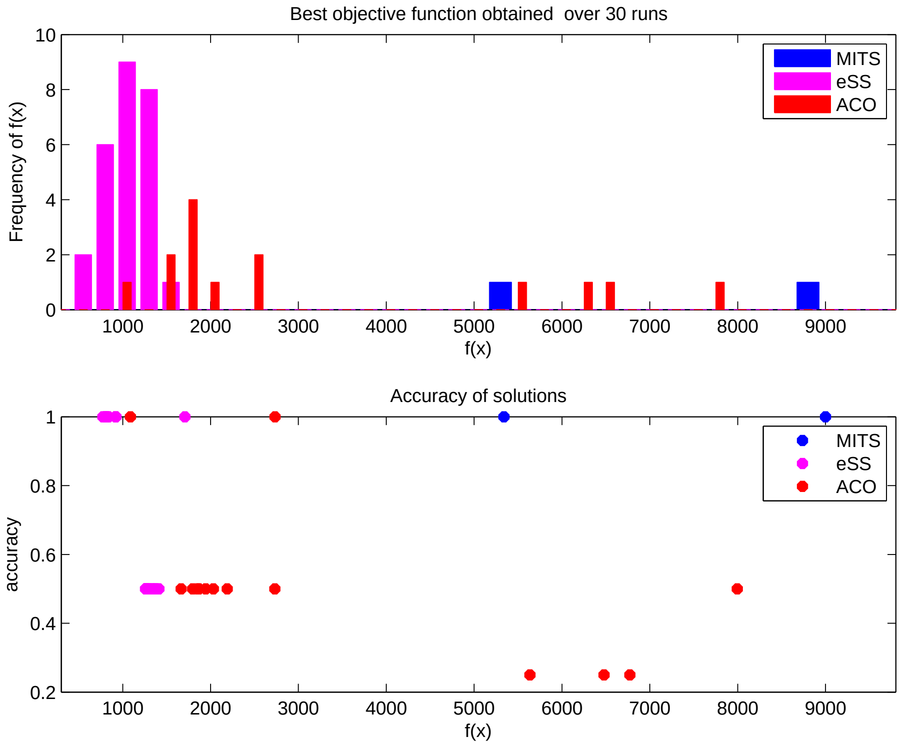

Figure S.11: *Case study 2 (E. coli homeostasis): Histogram of achieved objective function values and accuracy of solution for logic-based implementation of the KdpD/KdpE two-component system problem.* Upper figure: histogram with the frequency distribution of the solutions obtained with 3 solvers. Most solutions from ACOmi and MITS are excluded from this chart since these are far from the optimal point. Below, accuracy is plotted for each group of solutions as a function of the final objective function value. Accuracy was computed as as $(TP+TN)/(TP+TN+FP+FN)$ where TP is the number of true positive, TN the number of true negative, FP the number of false positive and FN the number of false negative links. As the correct solution is unknown for this case, the comparison is done against the best solution found.

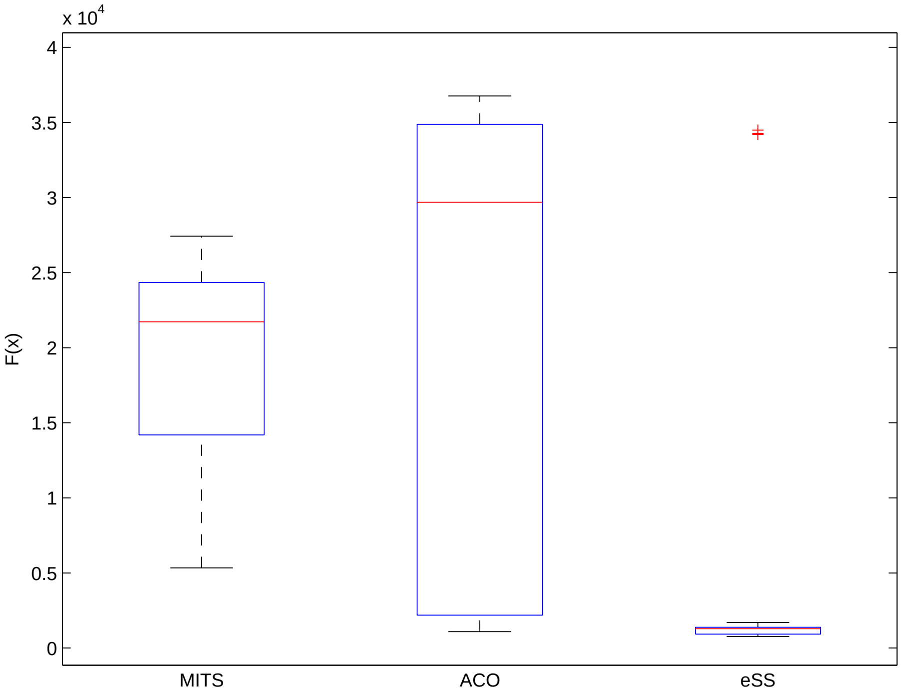

Figure S.12: *Case study 2 (E. coli homeostasis): Boxplot of the final objective function value for each optimization method (eSS, ACO, MITS) while solving the KdpD/KdpE two-component system problem.*

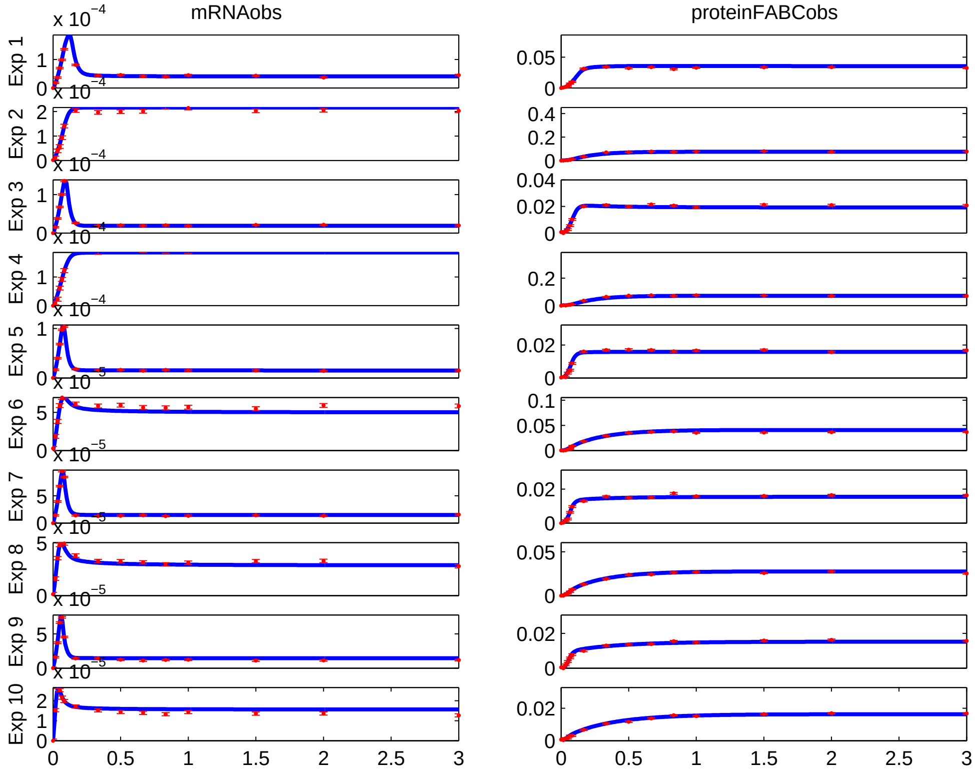

Figure S.13: *Case study 2 (E. coli homeostasis): Predicted versus observed time series for the best solution found.* The model described in [7] was used to generate pseudo-experimental data. Homoscedastic noise of 5% was added to this data (based on the mean of each experiment). Data points with error bars are shown in red while blue curves represent the simulation time-courses of the logic-based implementation of this problem. The agreement between the pseudo-experimental data and the calibrated logic-based model is very good.

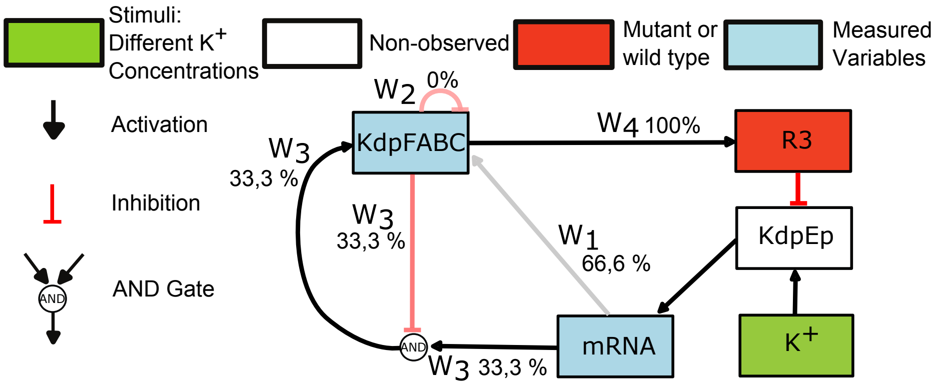

Figure S.14: *Case study 2 (E. coli homeostasis): Frequency of hyperedges in the model for near optimal solutions with objective function values under 1500.* The CellNOptR [8] software was used to illustrate specific hyperedges appear in near optimal solutions. In this example we consider solutions with an objective function value under 1500. Redundant hypereges were filtered after the optimization procedure. In this problem 4 binary variables were considered; w_1, w_2 , w_3 and w_4. The hyperedge w_4 is present in every of these solutions. On the other hand w_2 is always absent. The hyperedges w_1 and w_3 appear in some solutions.

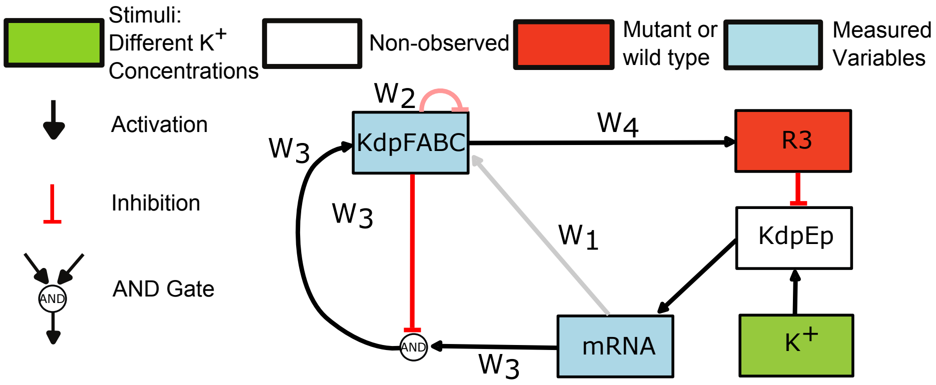

Figure S.15: *Case study 2 (E. coli homeostasis): Frequency of hyperedges in the model for near optimal solutions with objective function values under 1000.* The CellNOptR [8] software was used to illustrate specific hyperedges appear in near optimal solutions. In this example we consider solutions with an objective function value under 1000. Redundant hypereges were filtered after the optimization procedure. In this problem 4 binary variables were considered; w_1, w_2 , w_3 and w_4. The hyperedges w_3 and w_4 were present in every of the top performing solutions whilew_1 and w_2 were always absent.

	MITS(2.17e+004)	eSS(1.29e+003)	ACO(2.97e+004)
MITS(2.17e+004)	–	$H = 1, P = 1.11 \cdot 10^{-6}$	$H = 0, P = 0.631$
eSS(1.29e+003)	$H = 1, P = 1.11 \cdot 10^{-6}$	–	$H = 1, P = 7.69 \cdot 10^{-8}$
ACO(2.97e+004)	$H = 0, P = 0.631$	$H = 1, P = 7.69 \cdot 10^{-8}$	–

Table S.3: *Case study 2 (E. coli homeostasis): The Wilcoxon rank sum test applied to the distributions of final objective function values obtained with each pair of methods.* The null hypothesis (H=0) is that both distributions have the same median (under parentheses) with a given probability (P).

3 Case Study 3: Signaling application to transformed liver hepatocytes (HepG2)

In this section we provide a number of details and optimization statistics obtained while solving case study 3. The model and data used in this section are of public domain and can be downloaded from `http://www.ebi.ac.uk/~cokelaer/cellnopt/data/ExtLiverBMC2012.html#extliverbmc2012`.

We used the standard CellNoptR preprocessing to obtain a compressed version of the model. Data was normalized between 0 and 1 by rescaling. The MINLP solvers considered here are based in Matlab, thus in order to use these methods and to implement the NLP formulation of the problem we generated an R parser to generate a dynamic model in C and a configuration file for the AMIGO toolbox. All values for the inhibitors were set to 0.9 . All value for the stimuli were set to 0.65.

This section contains the following elements (in this order):

1. Experimental data for the HepG2 case study.
2. Stimuli and inhibitor values from experiments 1 to 32.
3. Stimuli and inhibitor values from experiments 33 to 64.
4. Original PKN before being compressed.
5. PKN network after compression.
6. Hypergraph after expansion.
7. Convergence curves for the HepG2 cell-line problem.
8. Convergence curves for the solution of the HepG2 problem using MPeSS.
9. Histogram of the final objective function value achieved by each optimizer.
10. Boxplot of the final objective function value achieved by each optimizer.

11. Frequency of hyperedges in the model for near optimal solutions with objective function values under 37.

12. Pareto front of the obtained solutions showing the complexity versus fitness trade-off.

13. Experimental versus predicted time series for each of the non-dominated solution.

14. Network structure for solution A (selected as best trade-off fit).

15. Network structure for solution B.

16. Network structure for solution C.

17. Network structure for solution D.

18. Network structure for solution E.

19. Network structure for solution F.

20. Data and simulation for solution A (experiments 1 to 16).

21. Data and simulation for solution A (experiments 17 to 32).

22. Data and simulation for solution A (experiments 33 to 48).

23. Data and simulation for solution A (experiments 49 to 64).

24. The Wilcoxon rank sum test for equal medians applied to the obtained distributions of final objective function values.

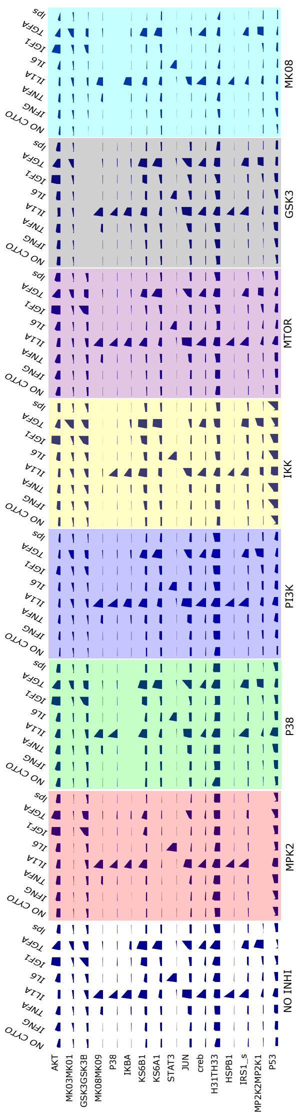

Figure S.16: *Case study 3 (HepG2): Experimental data for the HepG2 cell-line case study.*

	IFNG	TNFA	IL1A	IL6	IGF1	TGFA	lps	MP2K2	p38	pi3k	ikk	MTOR	GSK3	MK08
exp 1	0.00	0.00	0.00	0.00	0.00	0.00	0.00	0.00	0.00	0.00	0.00	0.00	0.00	0.00
exp 2	0.65	0.00	0.00	0.00	0.00	0.00	0.00	0.00	0.00	0.00	0.00	0.00	0.00	0.00
exp 3	0.00	0.65	0.00	0.00	0.00	0.00	0.00	0.00	0.00	0.00	0.00	0.00	0.00	0.00
exp 4	0.00	0.00	0.65	0.00	0.00	0.00	0.00	0.00	0.00	0.00	0.00	0.00	0.00	0.00
exp 5	0.00	0.00	0.00	0.65	0.00	0.00	0.00	0.00	0.00	0.00	0.00	0.00	0.00	0.00
exp 6	0.00	0.00	0.00	0.00	0.65	0.00	0.00	0.00	0.00	0.00	0.00	0.00	0.00	0.00
exp 7	0.00	0.00	0.00	0.00	0.00	0.65	0.00	0.00	0.00	0.00	0.00	0.00	0.00	0.00
exp 8	0.00	0.00	0.00	0.00	0.00	0.00	0.65	0.00	0.00	0.00	0.00	0.00	0.00	0.00
exp 9	0.00	0.00	0.00	0.00	0.00	0.00	0.00	0.90	0.00	0.00	0.00	0.00	0.00	0.00
exp 10	0.65	0.00	0.00	0.00	0.00	0.00	0.00	0.90	0.00	0.00	0.00	0.00	0.00	0.00
exp 11	0.00	0.65	0.00	0.00	0.00	0.00	0.00	0.90	0.00	0.00	0.00	0.00	0.00	0.00
exp 12	0.00	0.00	0.65	0.00	0.00	0.00	0.00	0.90	0.00	0.00	0.00	0.00	0.00	0.00
exp 13	0.00	0.00	0.00	0.65	0.00	0.00	0.00	0.90	0.00	0.00	0.00	0.00	0.00	0.00
exp 14	0.00	0.00	0.00	0.00	0.65	0.00	0.00	0.90	0.00	0.00	0.00	0.00	0.00	0.00
exp 15	0.00	0.00	0.00	0.00	0.00	0.65	0.00	0.90	0.00	0.00	0.00	0.00	0.00	0.00
exp 16	0.00	0.00	0.00	0.00	0.00	0.00	0.65	0.90	0.00	0.00	0.00	0.00	0.00	0.00
exp 17	0.00	0.00	0.00	0.00	0.00	0.00	0.00	0.00	0.90	0.00	0.00	0.00	0.00	0.00
exp 18.	0.65	0.00	0.00	0.00	0.00	0.00	0.00	0.00	0.90	0.00	0.00	0.00	0.00	0.00
exp 19	0.00	0.65	0.00	0.00	0.00	0.00	0.00	0.00	0.90	0.00	0.00	0.00	0.00	0.00
exp 20	0.00	0.00	0.65	0.00	0.00	0.00	0.00	0.00	0.90	0.00	0.00	0.00	0.00	0.00
exp 21	0.00	0.00	0.00	0.65	0.00	0.00	0.00	0.00	0.90	0.00	0.00	0.00	0.00	0.00
exp 22	0.00	0.00	0.00	0.00	0.65	0.00	0.00	0.00	0.90	0.00	0.00	0.00	0.00	0.00
exp 23	0.00	0.00	0.00	0.00	0.00	0.65	0.00	0.00	0.90	0.00	0.00	0.00	0.00	0.00
exp 24	0.00	0.00	0.00	0.00	0.00	0.00	0.65	0.00	0.90	0.00	0.00	0.00	0.00	0.00
exp 25	0.00	0.00	0.00	0.00	0.00	0.00	0.00	0.00	0.00	0.90	0.00	0.00	0.00	0.00
exp 26	0.65	0.00	0.00	0.00	0.00	0.00	0.00	0.00	0.00	0.90	0.00	0.00	0.00	0.00
exp 27	0.00	0.65	0.00	0.00	0.00	0.00	0.00	0.00	0.00	0.90	0.00	0.00	0.00	0.00
exp 28	0.00	0.00	0.65	0.00	0.00	0.00	0.00	0.00	0.00	0.90	0.00	0.00	0.00	0.00
exp 29	0.00	0.00	0.00	0.65	0.00	0.00	0.00	0.00	0.00	0.90	0.00	0.00	0.00	0.00
exp 30	0.00	0.00	0.00	0.00	0.65	0.00	0.00	0.00	0.00	0.90	0.00	0.00	0.00	0.00
exp 31	0.00	0.00	0.00	0.00	0.00	0.65	0.00	0.00	0.00	0.90	0.00	0.00	0.00	0.00
exp 32	0.00	0.00	0.00	0.00	0.00	0.00	0.65	0.00	0.00	0.90	0.00	0.00	0.00	0.00

Table S.4: Case study 3 (HepG2): Stimuli and inhibitor values for experiments 1 to 32.

	IFNG	TNFA	IL1A	IL6	IGF1	TGFA	lps	MP2K2	p38	pi3k	ikk	MTOR	GSK3	MK08
exp 33	0.00	0.00	0.00	0.00	0.00	0.00	0.00	0.00	0.00	0.00	0.90	0.00	0.00	0.00
exp 34	0.65	0.00	0.00	0.00	0.00	0.00	0.00	0.00	0.00	0.00	0.90	0.00	0.00	0.00
exp 35	0.00	0.65	0.00	0.00	0.00	0.00	0.00	0.00	0.00	0.00	0.90	0.00	0.00	0.00
exp 36	0.00	0.00	0.65	0.00	0.00	0.00	0.00	0.00	0.00	0.00	0.90	0.00	0.00	0.00
exp 37	0.00	0.00	0.00	0.65	0.00	0.00	0.00	0.00	0.00	0.00	0.90	0.00	0.00	0.00
exp 38	0.00	0.00	0.00	0.00	0.65	0.00	0.00	0.00	0.00	0.00	0.90	0.00	0.00	0.00
exp 39	0.00	0.00	0.00	0.00	0.00	0.65	0.00	0.00	0.00	0.00	0.90	0.00	0.00	0.00
exp 40	0.00	0.00	0.00	0.00	0.00	0.00	0.65	0.00	0.00	0.00	0.90	0.00	0.00	0.00
exp 41	0.00	0.00	0.00	0.00	0.00	0.00	0.00	0.00	0.00	0.00	0.00	0.90	0.00	0.00
exp 42	0.65	0.00	0.00	0.00	0.00	0.00	0.00	0.00	0.00	0.00	0.00	0.90	0.00	0.00
exp 43	0.00	0.65	0.00	0.00	0.00	0.00	0.00	0.00	0.00	0.00	0.00	0.90	0.00	0.00
exp 44	0.00	0.00	0.65	0.00	0.00	0.00	0.00	0.00	0.00	0.00	0.00	0.90	0.00	0.00
exp 45	0.00	0.00	0.00	0.65	0.00	0.00	0.00	0.00	0.00	0.00	0.00	0.90	0.00	0.00
exp 46	0.00	0.00	0.00	0.00	0.65	0.00	0.00	0.00	0.00	0.00	0.00	0.90	0.00	0.00
exp 47	0.00	0.00	0.00	0.00	0.00	0.65	0.00	0.00	0.00	0.00	0.00	0.90	0.00	0.00
exp 48	0.00	0.00	0.00	0.00	0.00	0.00	0.65	0.00	0.00	0.00	0.00	0.90	0.00	0.00
exp 49	0.00	0.00	0.00	0.00	0.00	0.00	0.00	0.00	0.00	0.00	0.00	0.00	0.90	0.00
exp 50	0.65	0.00	0.00	0.00	0.00	0.00	0.00	0.00	0.00	0.00	0.00	0.00	0.90	0.00
exp 51	0.00	0.65	0.00	0.00	0.00	0.00	0.00	0.00	0.00	0.00	0.00	0.00	0.90	0.00
exp 52	0.00	0.00	0.65	0.00	0.00	0.00	0.00	0.00	0.00	0.00	0.00	0.00	0.90	0.00
exp 53	0.00	0.00	0.00	0.65	0.00	0.00	0.00	0.00	0.00	0.00	0.00	0.00	0.90	0.00
exp 54	0.00	0.00	0.00	0.00	0.65	0.00	0.00	0.00	0.00	0.00	0.00	0.00	0.90	0.00
exp 55	0.00	0.00	0.00	0.00	0.00	0.65	0.00	0.00	0.00	0.00	0.00	0.00	0.90	0.00
exp 56	0.00	0.00	0.00	0.00	0.00	0.00	0.65	0.00	0.00	0.00	0.00	0.00	0.90	0.00
exp 57	0.00	0.00	0.00	0.00	0.00	0.00	0.00	0.00	0.00	0.00	0.00	0.00	0.00	0.90
exp 58	0.65	0.00	0.00	0.00	0.00	0.00	0.00	0.00	0.00	0.00	0.00	0.00	0.00	0.90
exp 59	0.00	0.65	0.00	0.00	0.00	0.00	0.00	0.00	0.00	0.00	0.00	0.00	0.00	0.90
exp 60	0.00	0.00	0.65	0.00	0.00	0.00	0.00	0.00	0.00	0.00	0.00	0.00	0.00	0.90
exp 61	0.00	0.00	0.00	0.65	0.00	0.00	0.00	0.00	0.00	0.00	0.00	0.00	0.00	0.90
exp 62	0.00	0.00	0.00	0.00	0.65	0.00	0.00	0.00	0.00	0.00	0.00	0.00	0.00	0.90
exp 63	0.00	0.00	0.00	0.00	0.00	0.65	0.00	0.00	0.00	0.00	0.00	0.00	0.00	0.90
exp 64	0.00	0.00	0.00	0.00	0.00	0.00	0.65	0.00	0.00	0.00	0.00	0.00	0.00	0.90

Table S.5: Case study 3 (HepG2): Stimuli and inhibitor values for experiments 33 to 64.

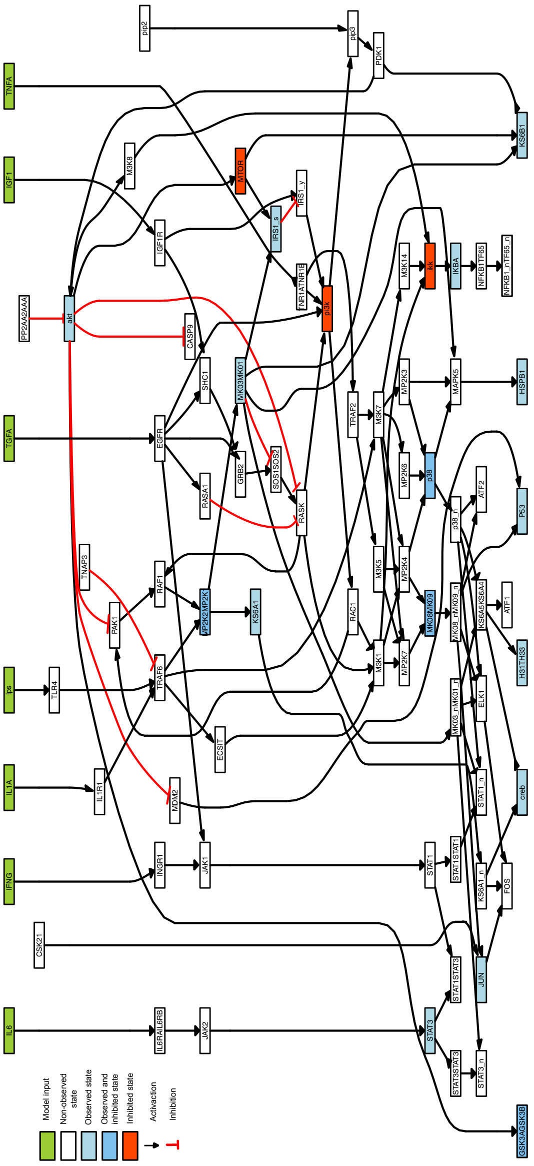

Figure S.17: *Case study 3 (HepG2): original PKN before being compressed* This figure was generated with the CellNOptR software.

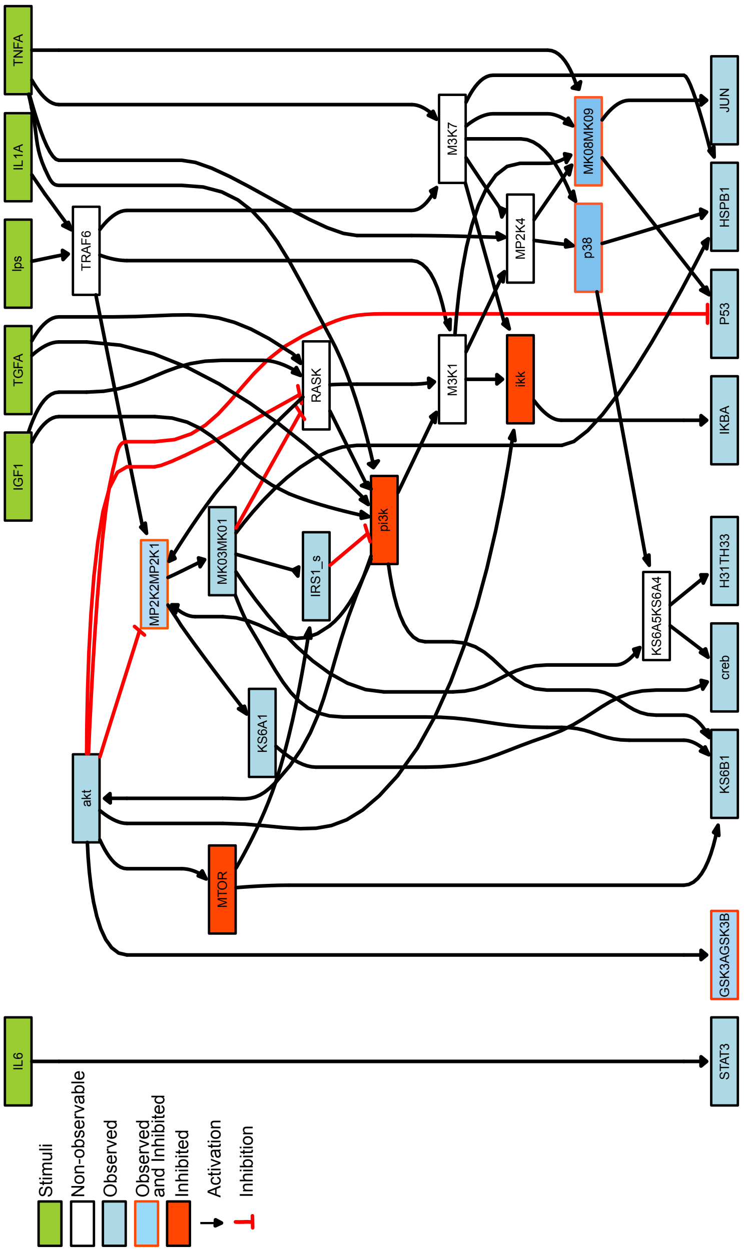

Figure S.18: *Case study 3 (HepG2): PKN network after compression.* Prior to expansion the model was compressed to remove non-observable/non-controllable species using CellNOptR[8] software.

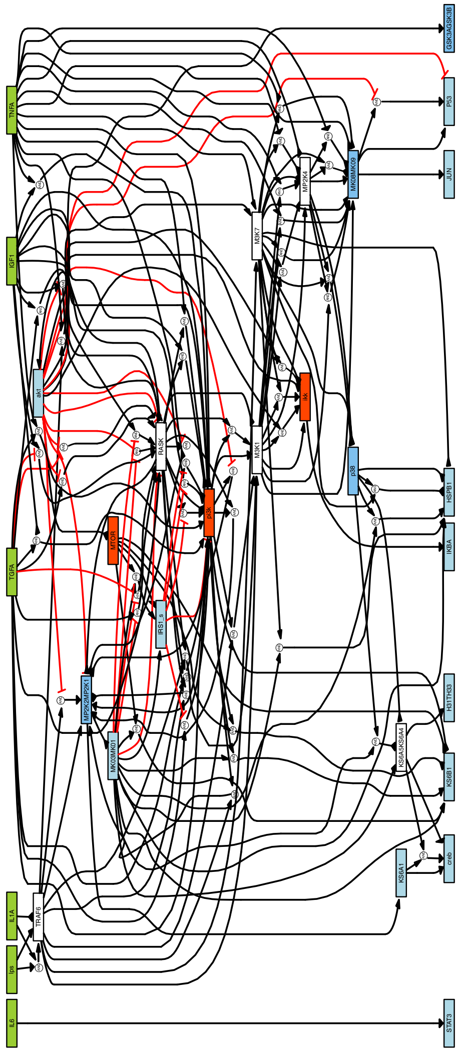

Figure S.19: *Case study 3 (HepG2): Hypergraph after expansion.* The original PKN was initially compressed to remove as many non-observable/non-controllable links as possible. Next the model was expanded to incorporate all possible hyperedges formed by two inputs. These derived in a model with 109 integer variables. The model was then used to formulate the MINLP problem containing 135 continuous parameters (n, k and τ), 109 binary variables (w) and 9 initial conditions.

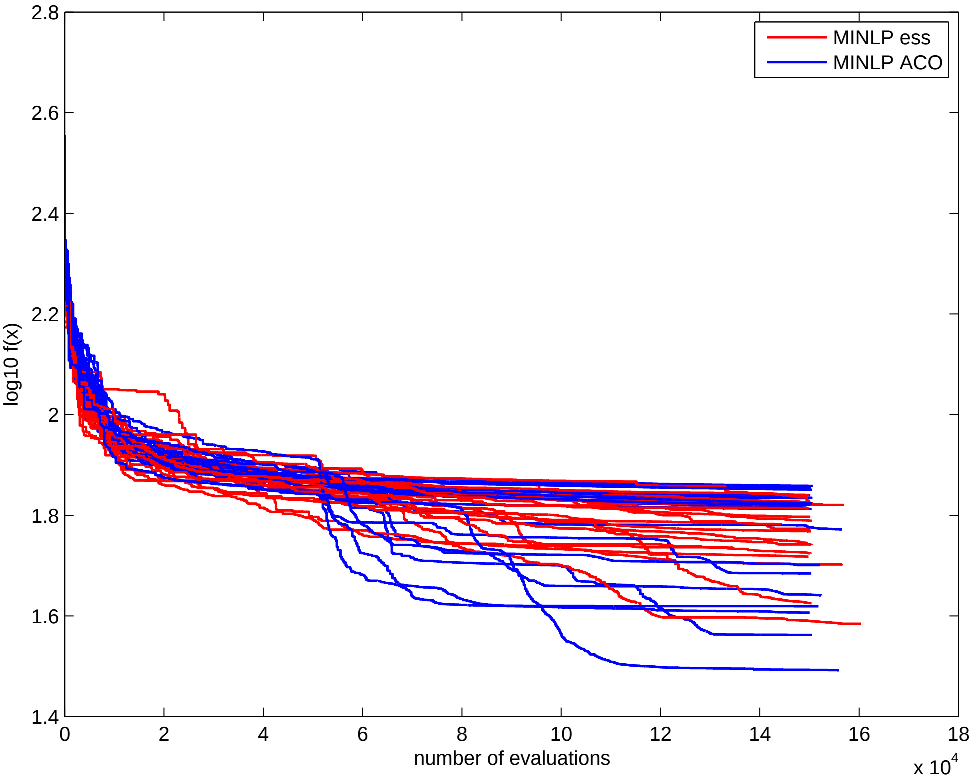

Figure S.20: *Case study 3 (HepG2): Convergence curves for the HepG2 cell-line problem using the MINLP formulation.* The MINLP problem was solved with 20 independent runs for each solver (eSS and ACOmi). No method was always able to solve the problem in the given function evaluation budget. Although ACOmi found the best solutions, its overall performance was comparable to that of eSS.

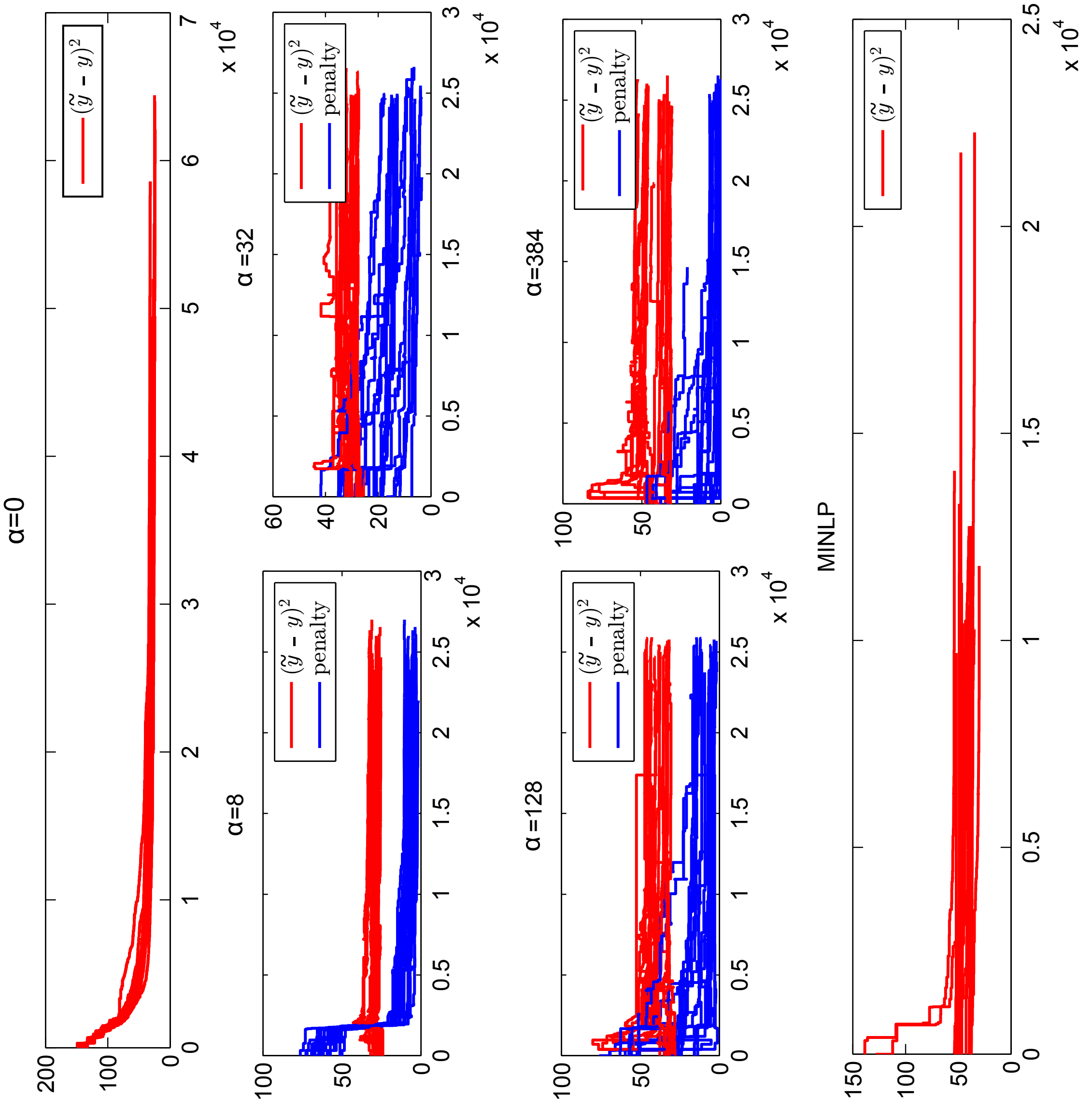

Figure S.21: *Case study 3 (HepG2): Convergence curves using MPeSS.* The optimization procedure was executed in 6 phases. In the first 5 phases we solved the relaxed problem with increasing values for α. In the final step, those solutions were used as initial points to solve the original MINLP formulation with eSS.

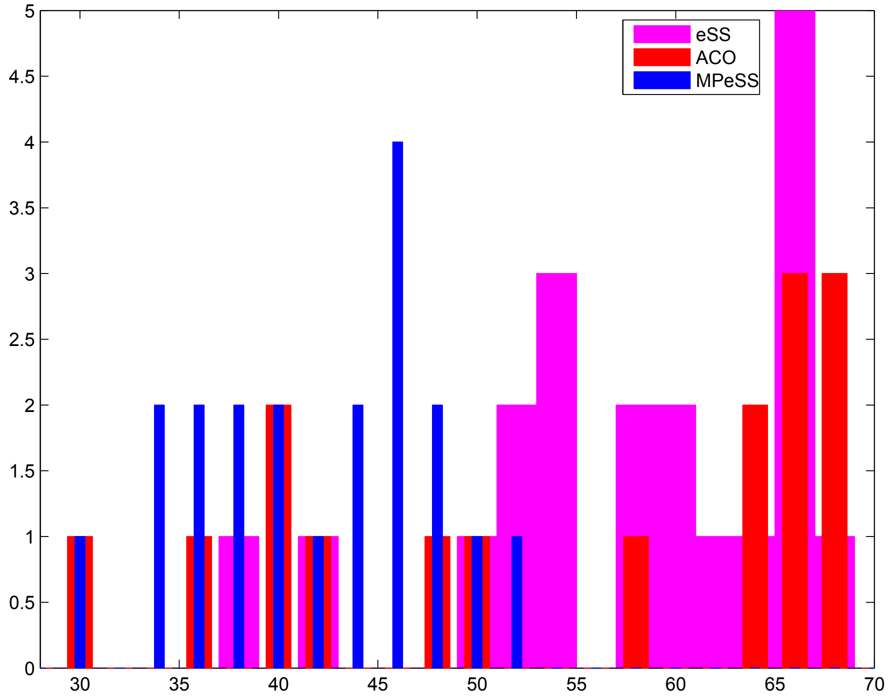

Figure S.22: *Case study 3 (HepG2): Histogram of the final objective function value achieved by each optimizer.* The problem was solved for 20 independent optimization runs with 3 solvers (eSS, MPeSS and ACOmi). All runs used the same budget of objective function evaluations.

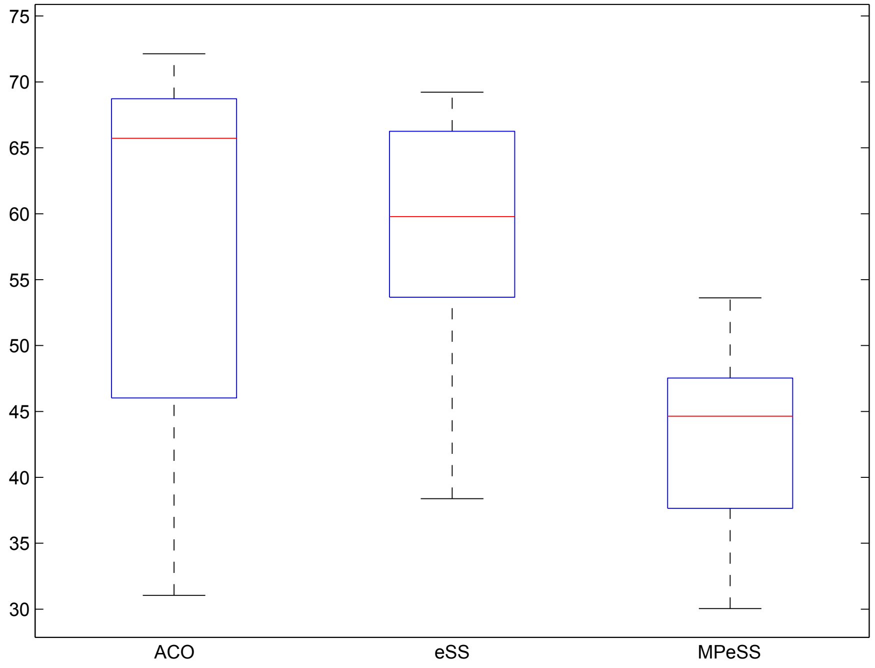

Figure S.23: *Case study 3 (HepG2): Boxplot of the final objective function value achieved by each optimizer (MPeSS, eSS, ACO).*

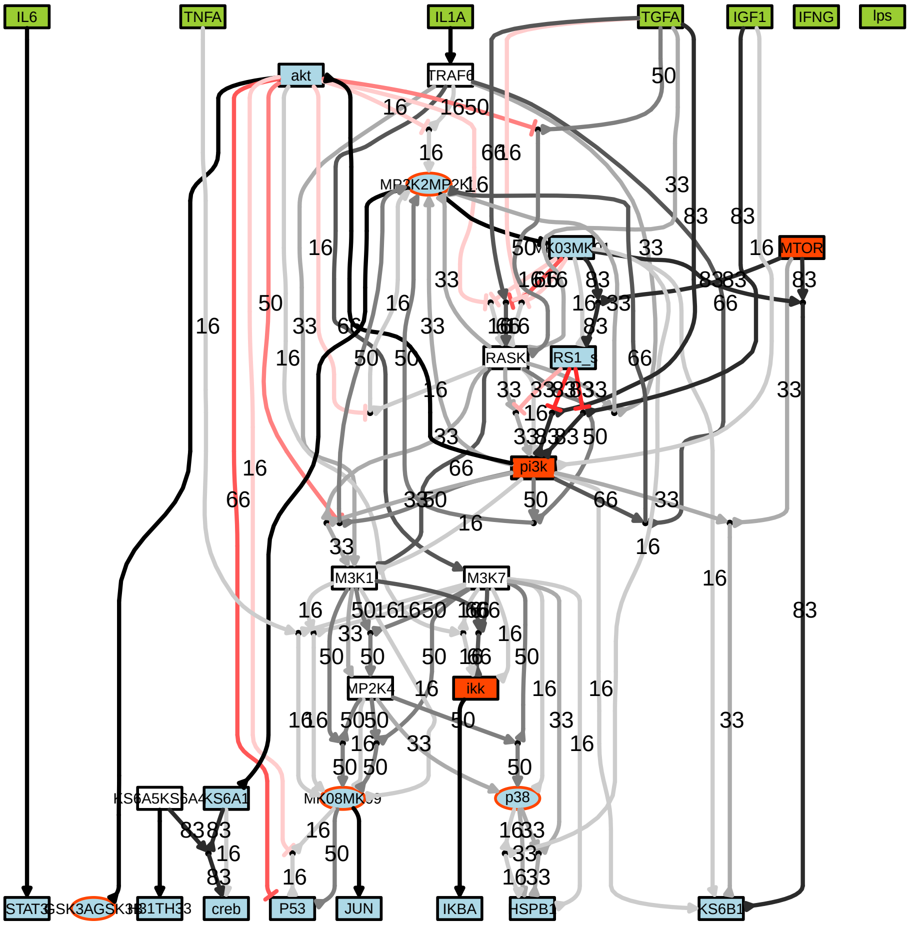

Figure S.24: *Case study 3 (HepG2): Frequency of hyperedges in the model for near optimal solutions with objective function values under 37.* The CellNOptR [8] software was used to illustrate how frequently certain hyperedges appear in near optimal solutions. In this example we consider solutions with an objective function value bellow 37 (a total of 6). Redundant hypereges were filtered after the optimization procedure. The numbers stand for the percentage each hyperedge appear in the final solutions. Strong dark or red links without any numbering illustrate links that are always present.

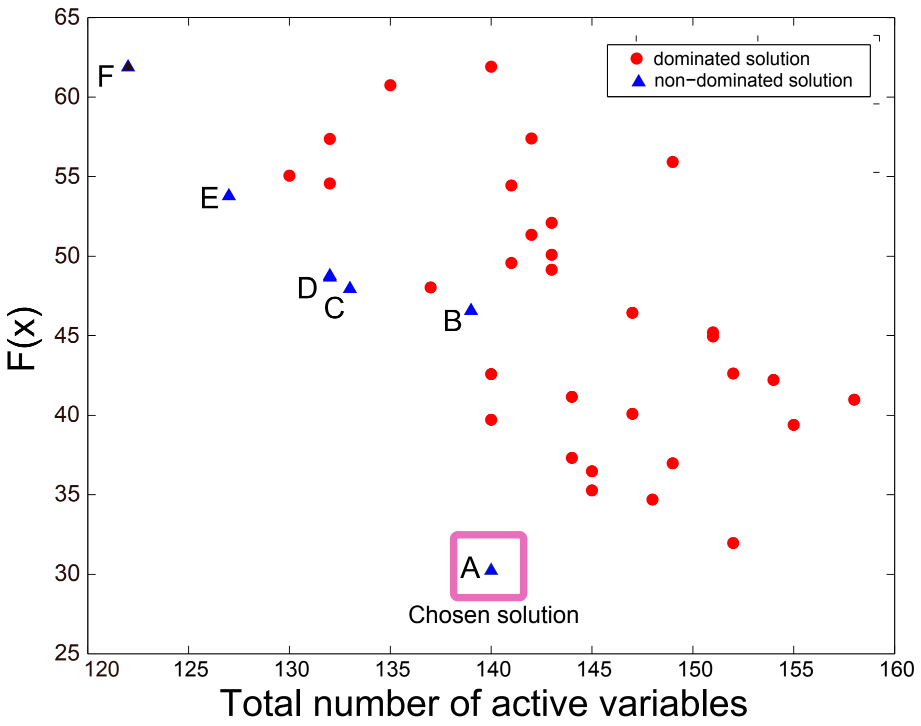

Figure S.25: *Case study 3 (HepG2): Pareto front of the obtained solutions, showing the trade-off between complexity and fit.* This figure shows the trade-off between fit (cost function) obtained by each independent optimization run and the number of active variables (number of active binary variables plus the number of active continuous parameters) which is a proxy for model complexity. The chosen solution shows the best trade-off between fitness and complexity.

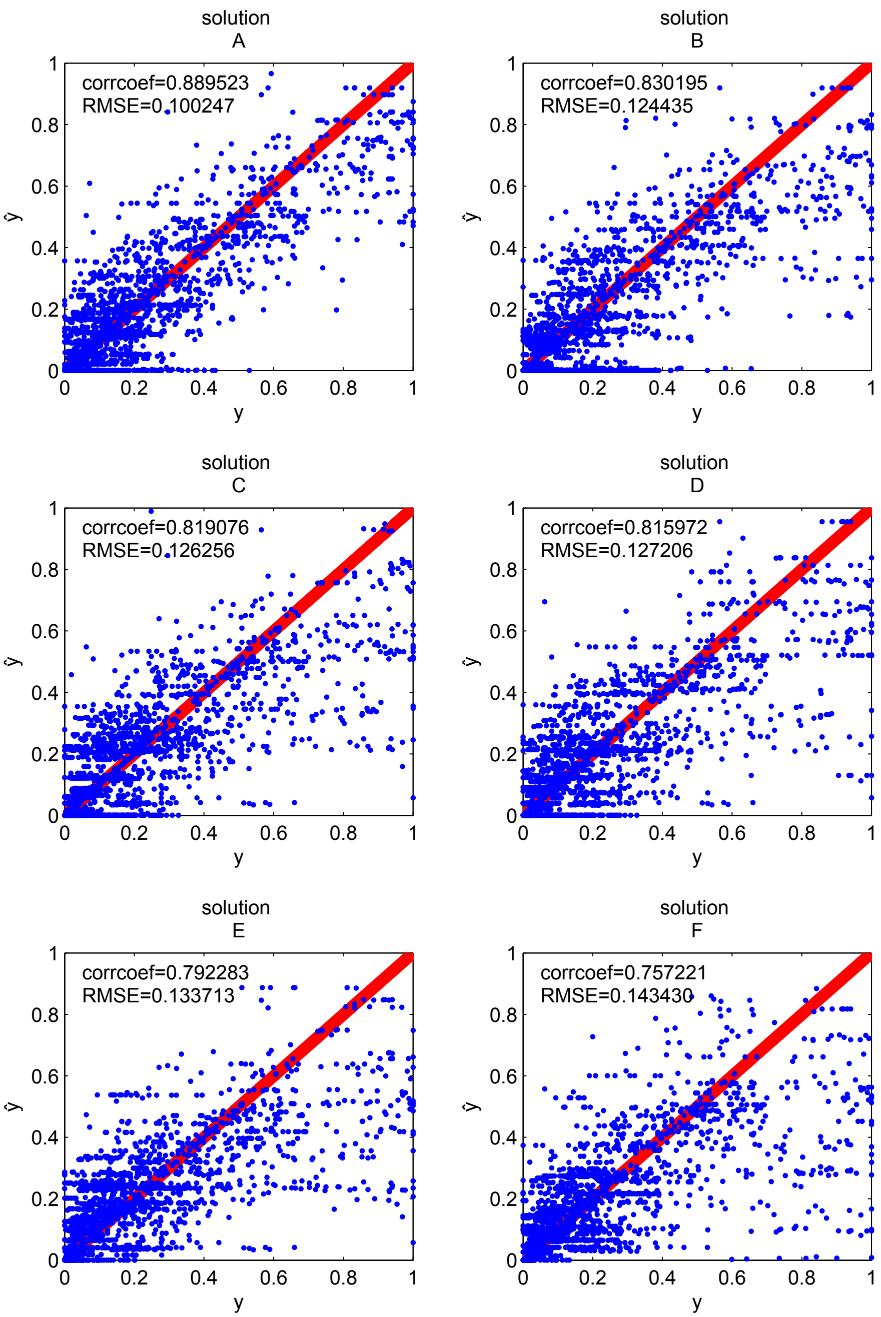

Figure S.26: *Case study 3 (HepG2): Experimental versus predicted data points for each of the non-dominated solution.* For each of the non-dominated solutions shown in figure S.25 the predicted values (by simulation) are plotted versus the experimental data. Correlation coefficients and root mean squared error values are also given to quantify the differences in the model fitness and/or potential overfitting.

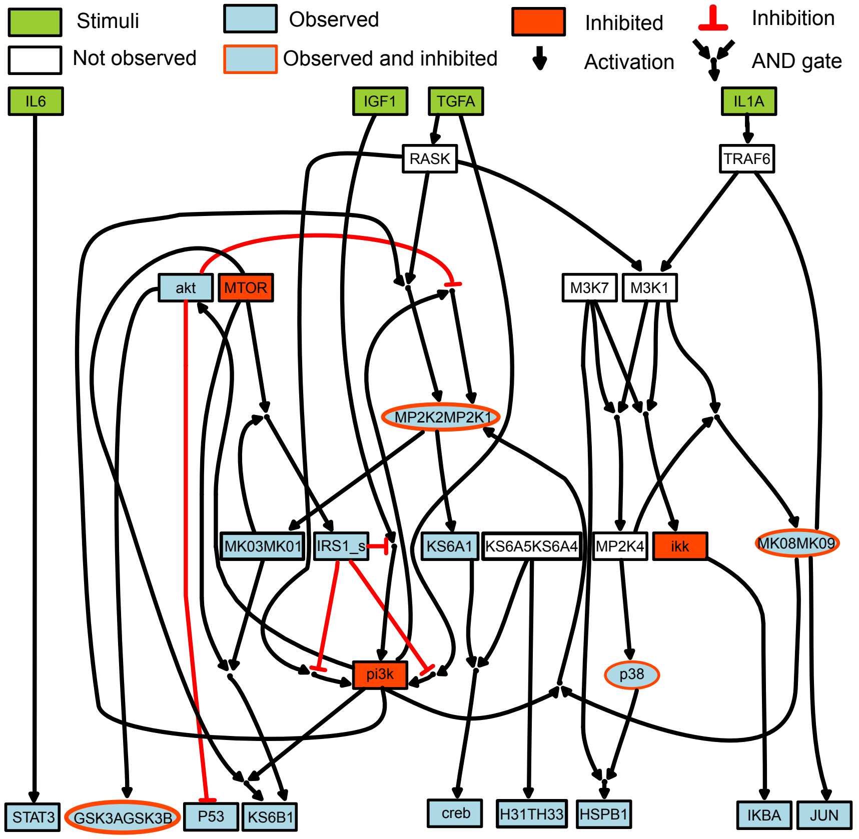

Figure S.27: *Case study 3 (HepG2): Network structure for solution A (best trrade-off).* Redundant hyperedges were removed from the model after the optimization procedure.

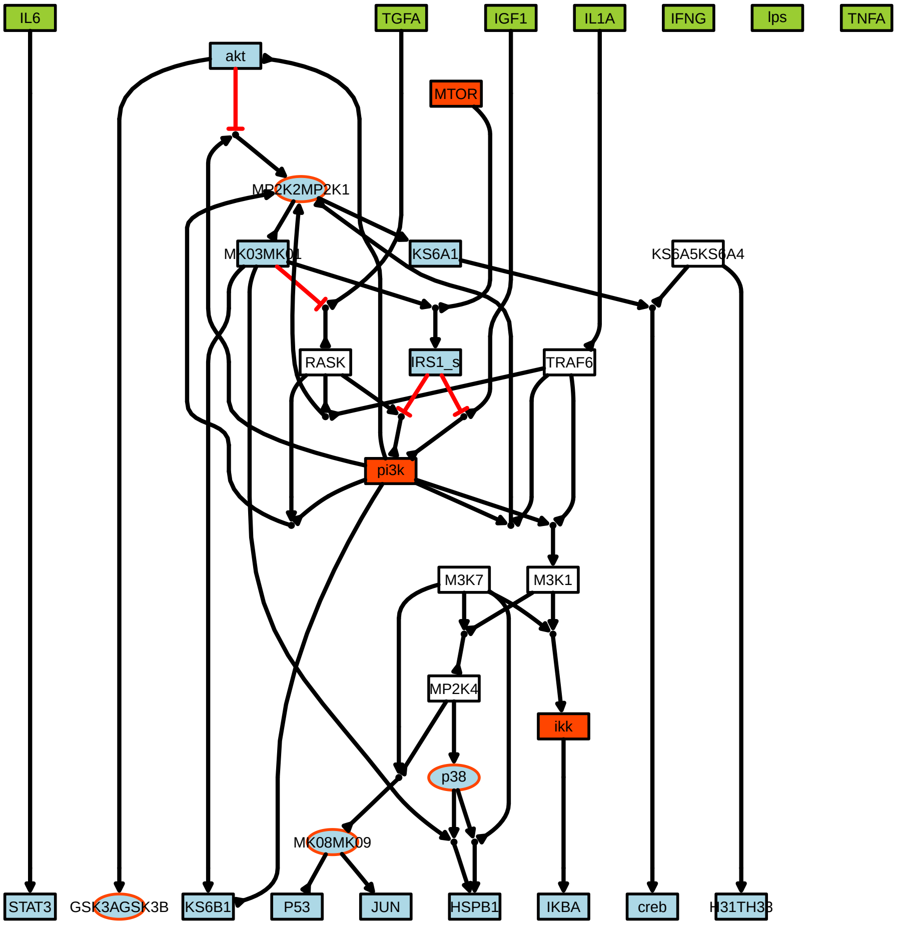

Figure S.28: *Case study 3 (HepG2): Network structure for solution B.* Redundant hyperedges were removed from the model after the optimization procedure.

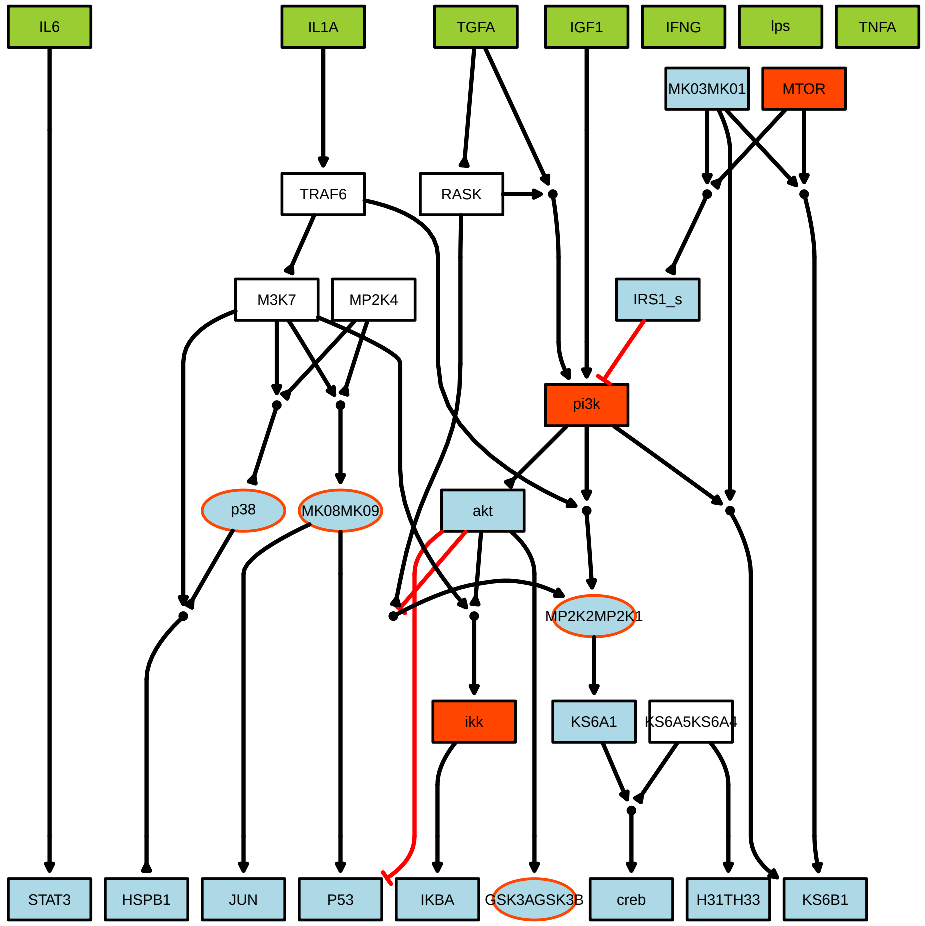

Figure S.29: *Case study 3 (HepG2): Network structure for solution C.* Redundant hyperedges were removed from the model after the optimization procedure.

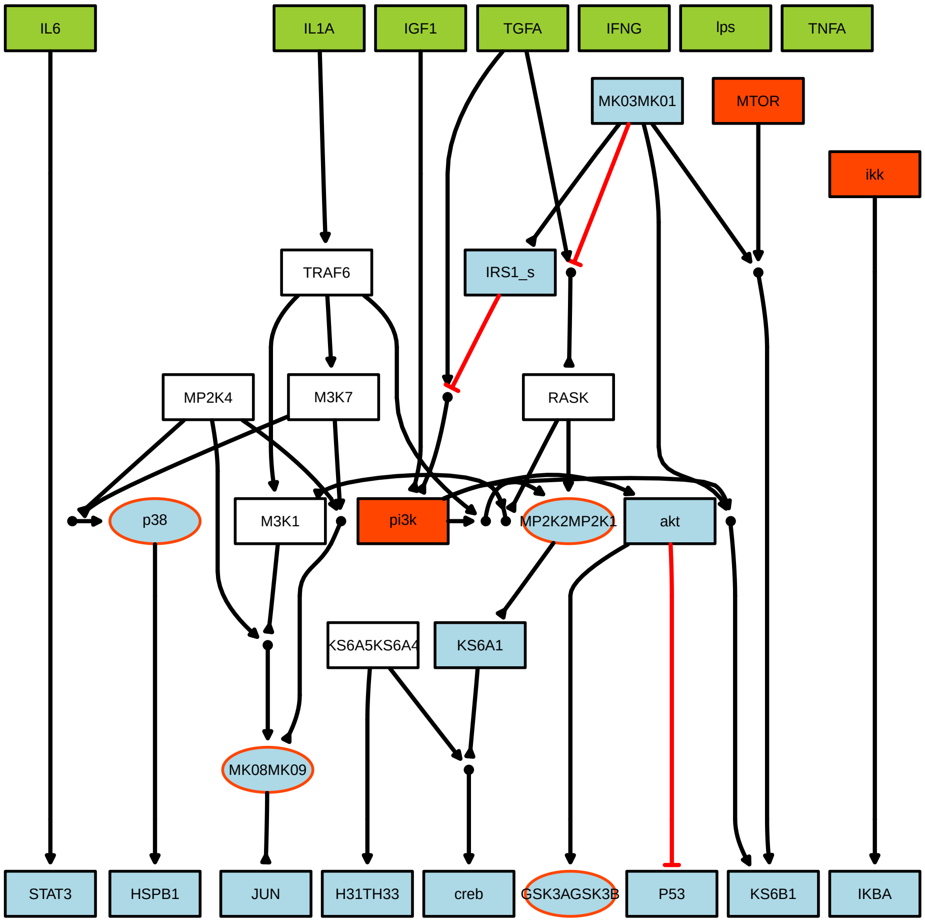

Figure S.30: *Case study 3 (HepG2): Network structure for solution D.* Redundant hyperedges were removed from the model after the optimization procedure.

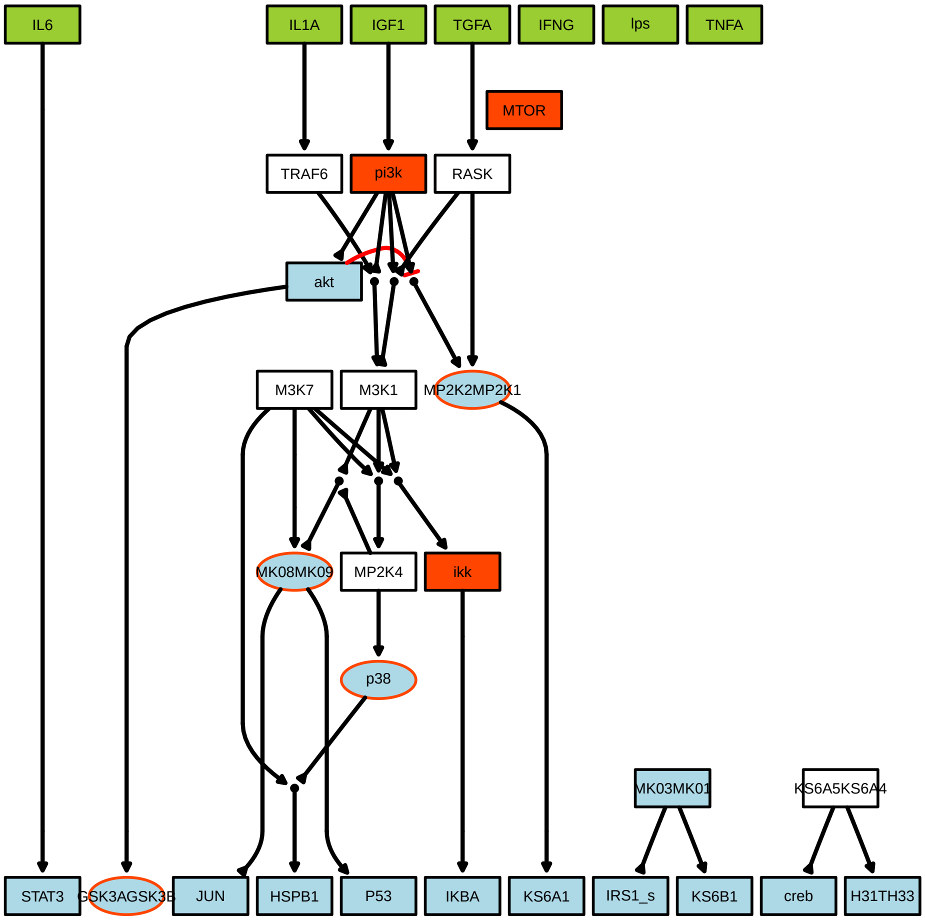

Figure S.31: *Case study 3 (HepG2): Network structure for solution E.* Redundant hyperedges were removed from the model after the optimization procedure.

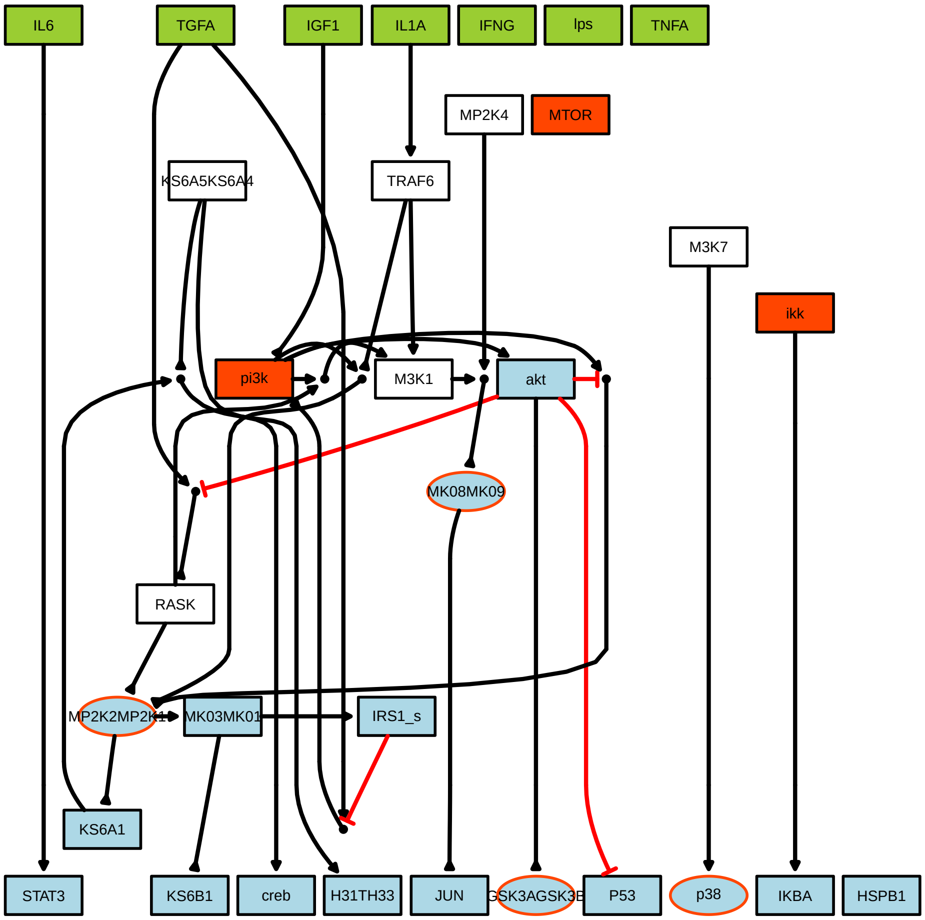

Figure S.32: *Case study 3 (HepG2): Network structure for solution F.* Redundant hyperedges were removed from the model after the optimization procedure.

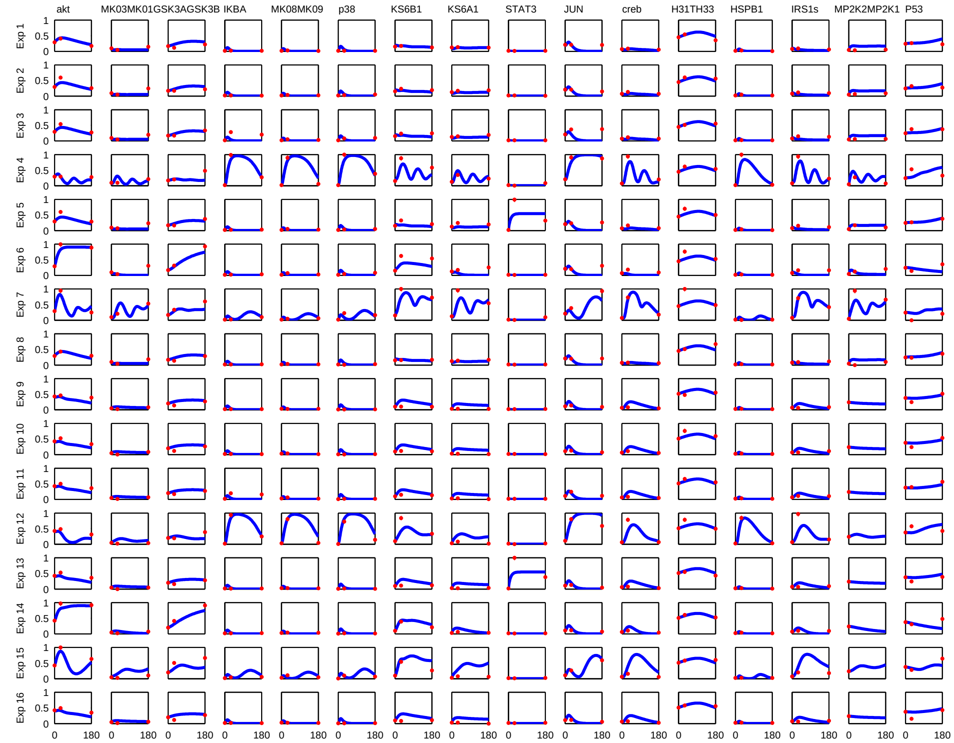

Figure S.33: *Case study 3 (HepG2): Data and model predictions for solution A (experiments 1 to 16).*

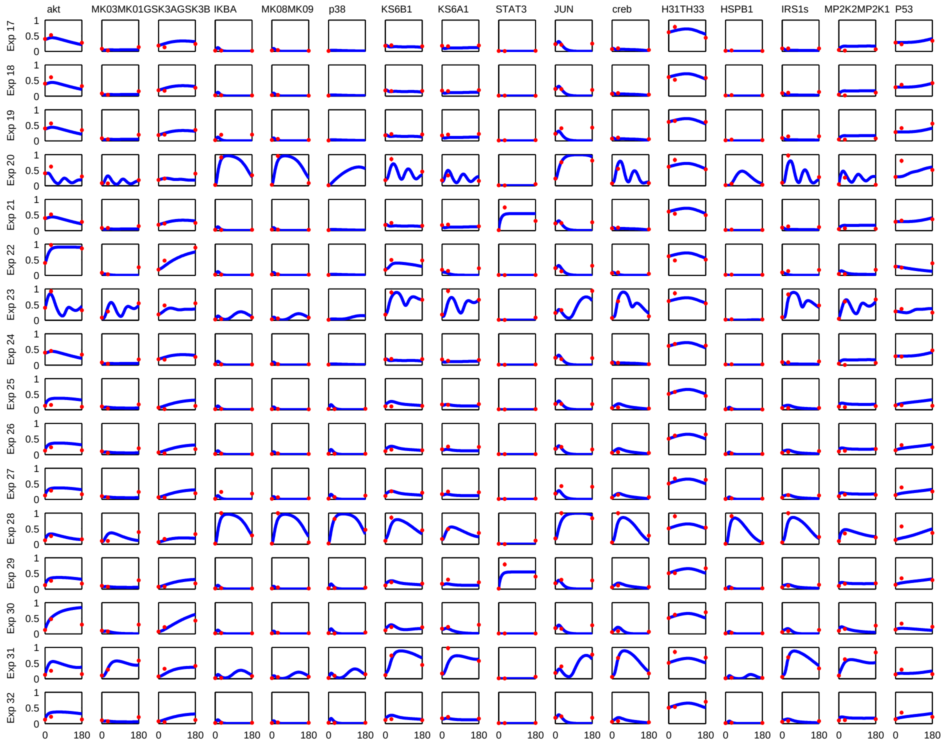

Figure S.34: Case study 3 (HepG2): Data and model predictions for solution A (experiments 17 to 32).

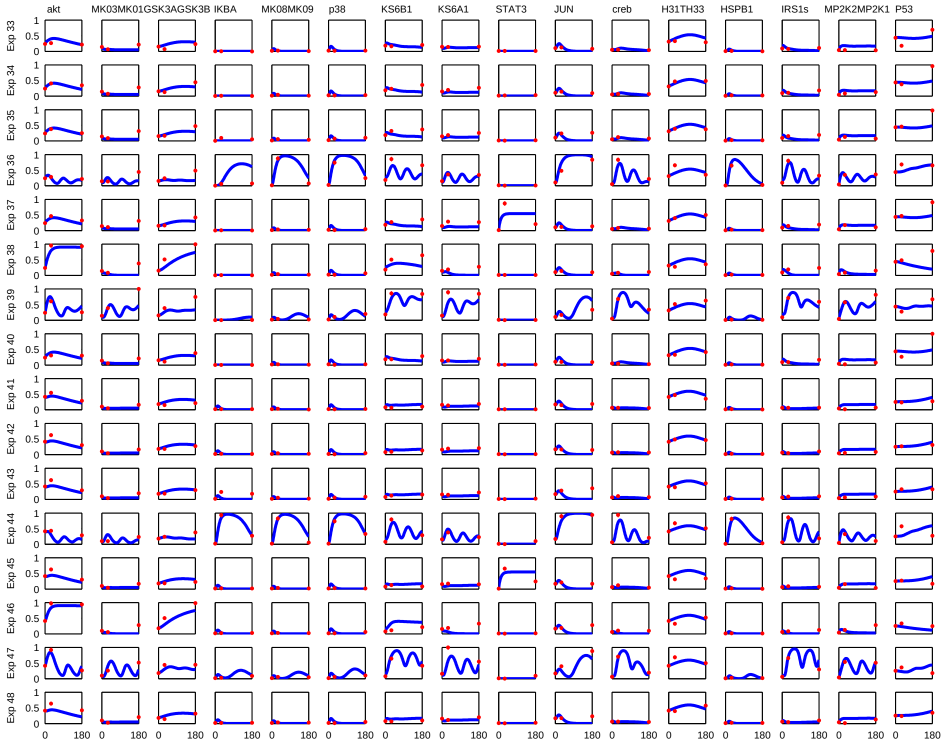

Figure S.35: *Case study 3 (HepG2): Data and model predictions for solution A (experiments 33 to 48).*

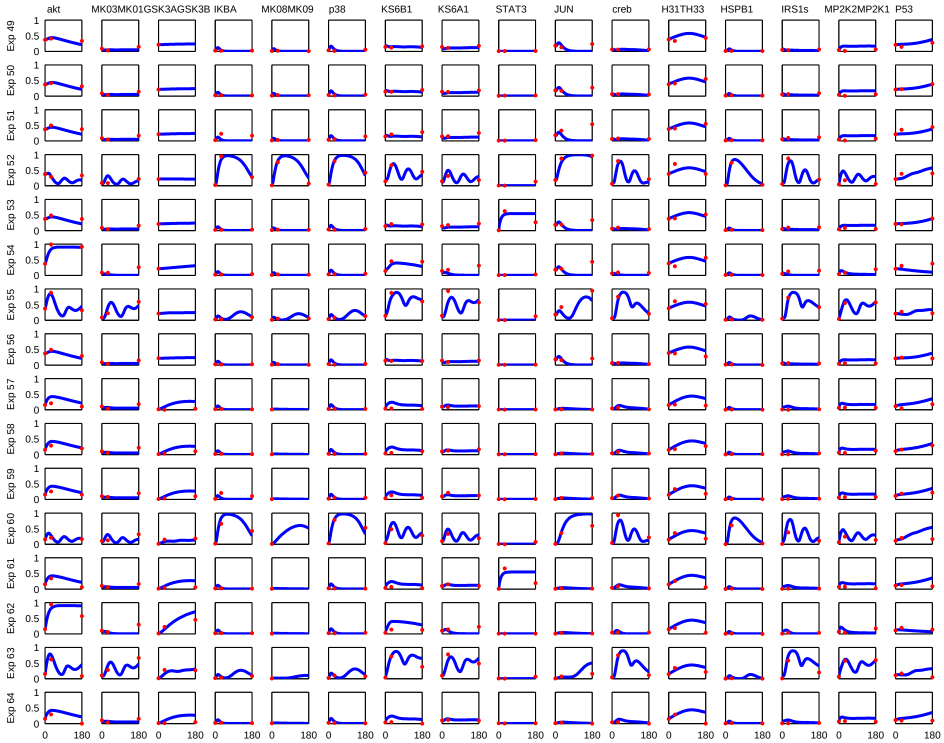

Figure S.36: *Case study 3 (HepG2): Data and model predictions for solution A (experiments 49 to 64).*

	eSS(59.8)	ACO(65.7)	MPeSS(44.6)
eSS(59.8)	–	$H = 0, P = 0.457$	$H = 1, P = 4.54 \cdot 10^{-6}$
ACO(65.7)	$H = 0, P = 0.457$	–	$H = 1, P = 8.36 \cdot 10^{-4}$
MPeSS(44.6)	$H = 1, P = 4.54 \cdot 10^{-6}$	$H = 1, P = 8.36 \cdot 10^{-4}$	–

Table S.6: *Case study 3 (HepG2): The Wilcoxon rank sum test applied to the distributions of final objective function values obtained with each pair of methods.* The null hypothesis (H=0) is that both distributions have the same median (under parentheses) with a given probability (P).

4 A brief comparison with the two time points Boolean approach

In this section we present a short comparison between results obtained with the MIDO approach we propose in this work and a Boolean logic method designed to capture two time points. The main assumption behind the two time points approach is that the system has two different pseudo steady-states. The first steady-state is governed by fast acting regulation mechanisms while the second is a consequence of later events.

¿From the optimization point of view the two time points model calibration is treated as two different optimization problems. The identification of the fast acting mechanism is done by finding the simplest logic model which explains the data at time point one. The links excluded at the first time point optimization are then considered in a second optimization problem.

Therefore we have two models. The first explains how the steady-state at time point one is reached and the second explains how the model prediction moves to the steady-state at the second time-point. The first model is therefore a subset of the second.

The advantage of using logic based ODEs becomes evident, i.e. the ability to handle time properly in such way that transient responses can be represented without the need of assumptions like having multiple steady-states. On the other hand, we no longer have a qualitative/Boolean model with all its advantages regarding analysis, interpretation and calibration.

4.1 The synthetic pathway model

The original goal of the synthetic pathway way model was to illustrate the differences of different formalisms related to logic models. The comparison shown in this section is similar to what is presented in [6] both at the conceptual level and the level of the used network.

Figure S.37 shows the compressed version of the synthetic pathway model the solution found by the two-time points algorithm in CellNOptR. Figure S.38 shows fitness of such solution. We can relate this plot to what is shown in Figure S.9, the fit for a solution found with the MIDO approach, showing that we were able to recover the full system dynamics. Except for p38 and and nfkb and p38, the Boolean model does a great job capturing the qualitative features of the system. In the case of nfkb the pseudo-experimental data incorporates oscillatory behavior which escapes the scope the two time time points approach.

This comparison is unfair in the sense that the data was generated with a logic-based ODE model which is included in the search space for the MIDO approach and we knew *a priori* that the Boolean model would not be able to grasp every detail of the simulated data. However the sole purpose of this analysis is to illustrate the need of using a dynamic model representation to describe certain model features.

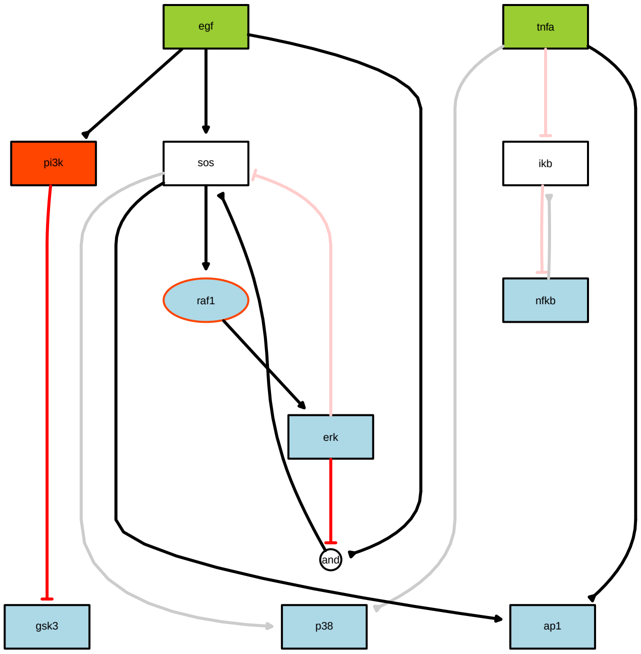

Figure S.37: *The Boolean model found by the two time points approach in the synthetic pathway case study* The CellNOptR software was used to calibrate the PKN to the pseudo-experimental data using the two time points strategy. The best performing model found is shown in this figure.

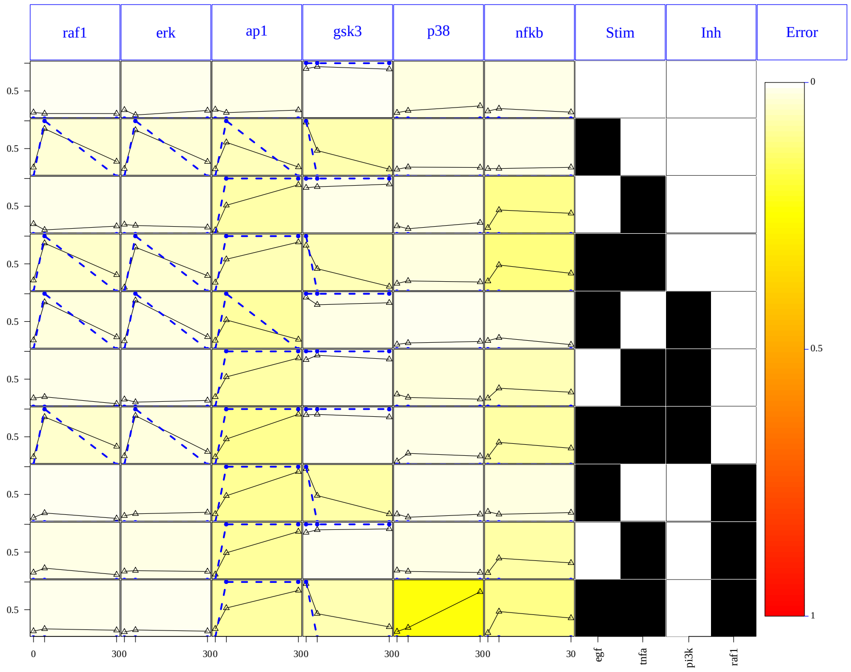

Figure S.38: *Fitness of the best Boolean model found in the synthetic pathway case study* The fitness of the best Boolean model found is shown for all observed species.

4.2 The HepG2 model

The human liver cancer cell-line problem poses an interesting case to apply both methods. The figures bellow are important to follow this analysis:

- Figure S.16: visualization of the data by combinations of perturbations.
- Figure S.18: The compressed PKN, describes the interactions considered by the optimization procedure.
- Figures S.33 to S.36 :the dynamic simulation of the model found with MIDO approach.
- Figure S.27: The best model found with the MIDO approach.
- Figure S.39: The best model found by the two time points approach.
- Figures S.40 and S.41: The Boolean logic simulation of the best model found with the two-time points approach.

Since we do not know the correct solution, analyzing both results can help finding anomalies caused by simulation artifacts. Consider for instance the following case:

- akt → GSK3A/GSK3B3: The Boolean model predicts that the activation of GSK3A/GSK3B3 is the consequence of a later event. However by inspecting the data we notice that GSK3A/GSK3B3 is slowly rising from the beginning (see experiments 22,30 and 28 for example).

On the other hand, particular cases where the quantitative model is able to describe the experimental data well and the qualitative model grossly fails deserve closer inspection. Two examples are given bellow:

- MK08MK09 → JUN: The Boolean model is incapable of explaining the behavior of JUN. The only option present in the PKN is that JUN in controlled only by MK08MK09. The data for MK08MK09 shows a fast transient after IL1A stimulation which the ODE model is able to reproduce well. However upon stimulation with TGFA the data for MK08/Mk09 does not show any transient behavior in contrast to JUN data. The ODE model is still able to find a good fit for MK08/MK09 and JUN. The explanation given by the ODE model is that there is a later transient behavior which is not visible in the data because we are not measuring the correct time points. Although this seems rather unreasonable it would easy to (in)validate this hypothesis by measuring intermediate time-points. A more reasonable explanation is that the PKN is missing an interaction. By inspecting the pathway hsa05200 (pathways in cancer) in Kegg [3] we see that MP2K1/MP2K2 (MEK) phosphorylates ERK which in turn phosphorylates JUN (c-JUN). The addition of this links should explain the behavior of JUN upon TGFA stimulation.

- Serine phophorylated IRS1: The Boolean models fails to explain IRS1_s behavior. The ODE model does reasonable job explaining IRS1 serine phophorylation which is mainly defined by MK03/Mk01. However we observed that IRS1_s is extremely sensitive. The data shows that in several occasions (see experiments 4,15,20,28, 36 for example) that MK03/MK01 is mildly activated without support from the experimental data. Again we turned to Kegg in order to find possible missing interactions in the PKN. A pathway for adypocites, hepatocytes and skeletal muscle cells of type II *diabetes mellitus* (ko04930) links the phosphorylation of the serine residue of IRS1 to mTOR, IKK and JNK(MPK08/MK09). Neither IKK and mTOR are measured. When these are inhibited we see a small (yet visible) effect on IRS1 upon stimulation with IL1A (see Figure S.16). For the case of MK08/MK09(JNK) we can directly contrast the model and data. The addition of an OR gate between MK08MK09 and IRS1, should be enough to correct the in Boolean logic case mismatch. Moreover the inhibition of MK08/MK09 causes a clear decrease in the IRS1_s upon IL1A stimulation.

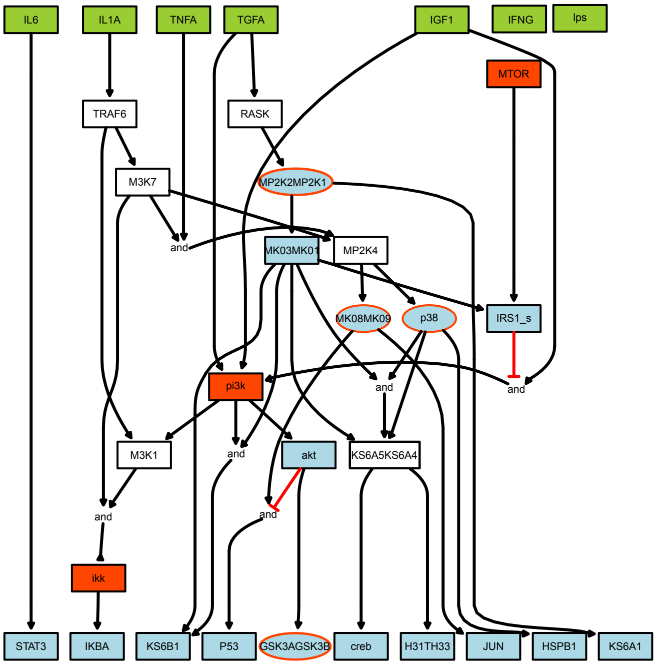

Figure S.39: *The Boolean model found by the two time points approach in the HepG2 case study* The CellNOptR software was used to calibrate the signaling pathway data to the human hepatocytes data using the two time points strategy. The best performing model found is shown in this figure.

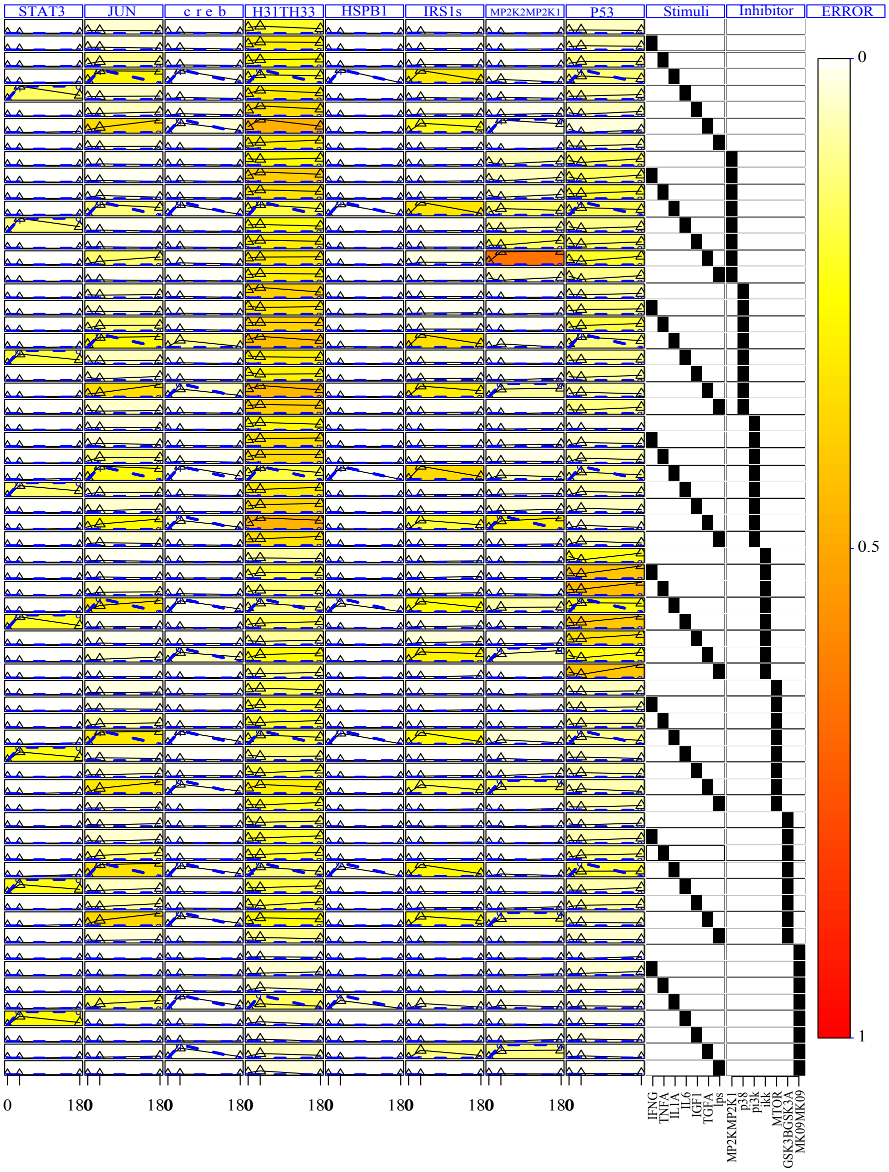

Figure S.40: *Fit of the best Boolean model found in the HepG2 case study (observables 1 to 8).* The fit of the best Boolean model found is shown for STAT3, JUN, creb, H31TH33, HSPB1, IRS1s, MP2K2MP2K1, and P53.

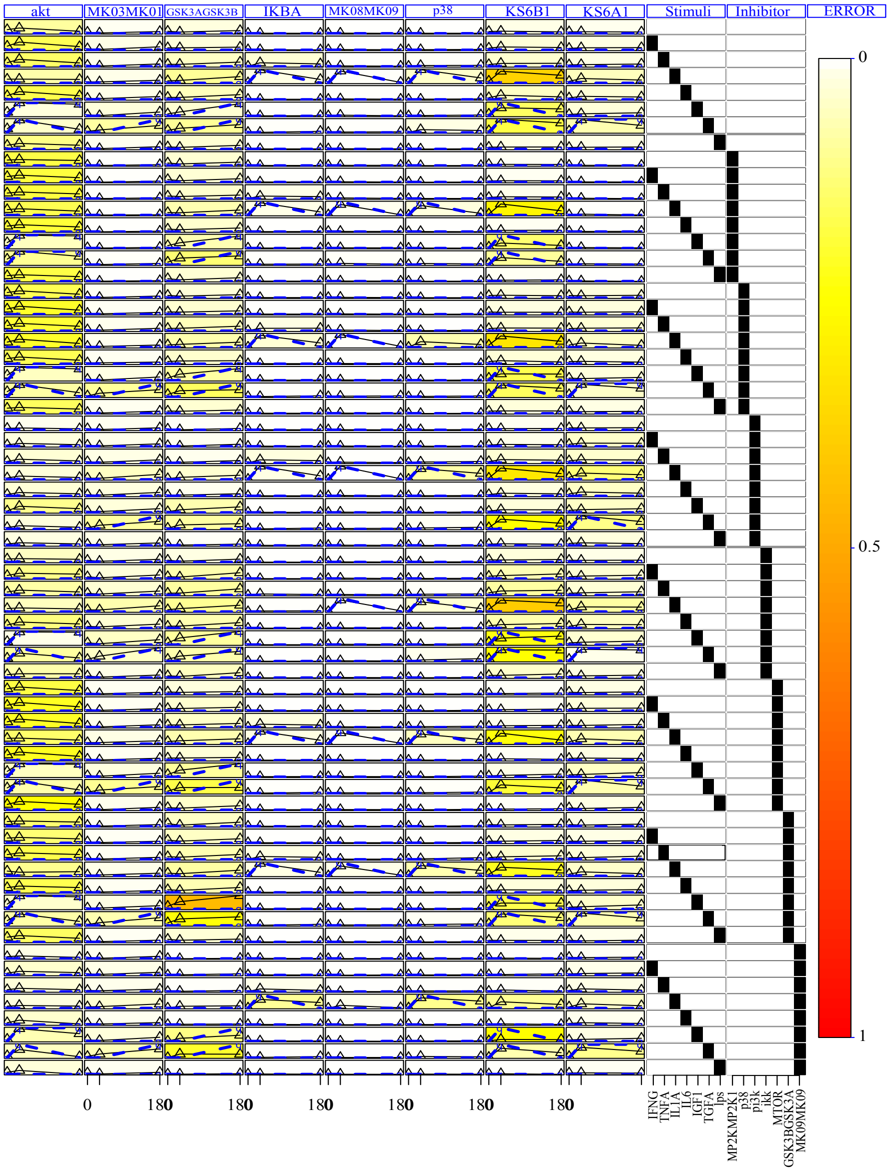

Figure S.41: *Fit of the best Boolean model found in the HepG2 case study (observables 9 to 16)* The fit of the best Boolean model found is shown for akt, MK03MK01, GSK3GSK3B, MK08MK09, p38, KS6B1 and KS6A1.

References

[1] Ralf Heermann and Kirsten Jung. The complexity of the simpletwo-component system kdpd/kdpe in escherichia coli. *FEMS microbiology letters*, 304(2):97–106, 2010.

[2] Kirsten Jung, Luitpold Fried, Stefan Behr, and Ralf Heermann. Histidine kinases and response regulators in networks. *Current opinion in microbiology*, 15(2):118–124, 2012.

[3] Minoru Kanehisa and Susumu Goto. Kegg: kyoto encyclopedia of genes and genomes. *Nucleic acids research*, 28(1):27–30, 2000.

[4] A Kremling, R Heermann, F Centler, K Jung, and ED Gilles. Analysis of two-component signal transduction by mathematical modeling using the *KdpD/KdpE* system of *Escherichia coli*. *Biosystems*, 78(1):23–37, 2004.

[5] Vera Laermann, Emina Ćudić, Kerstin Kipschull, Petra Zimmann, and Karl-heinz Altendorf. The sensor kinase kdpd of escherichia coli senses external k+. *Molecular microbiology*, 2013.

[6] Aidan MacNamara, Camille Terfve, David Henriques, Beatriz Peñalver Bernabé, and Julio Saez-Rodriguez. State–time spectrum of signal transduction logic models. *Physical Biology*, 9(4):045003, 2012.

[7] Maria Rodriguez-Fernandez, Markus Rehberg, Andreas Kremling, and Julio R Banga. Simultaneous model discrimination and parameter estimation in dynamic models of cellular systems. *BMC systems biology*, 7:76, 2013.

[8] Camille Terfve, Thomas Cokelaer, David Henriques, Aidan MacNamara, Emanuel Goncalves, Melody K Morris, Martijn van Iersel, Douglas A Lauffenburger, and J Saez-Rodrigues. Cellnoptr: a flexible toolkit to train protein signaling networks to data using multiple logic formalisms. *BMC Syst Biol*, 6(1):133, 2012.
